# Simplified cloning and isolation of peptides from “sandwiched” SUMO-peptide-intein fusion proteins

**DOI:** 10.1186/s12896-023-00779-5

**Published:** 2023-04-05

**Authors:** Tess Lamer, John C. Vederas

**Affiliations:** Department of Chemistry, University of Alberta, Edmonton, AB T6G 2G2 Canada

**Keywords:** Peptide, Bacteriocin, Heterologous expression, Small ubiquitin-like modifier (SUMO), Intein, Protein purification, Fusion protein, *Escherichia coli*

## Abstract

**Background:**

Some peptides are targets for degradation when heterologously expressed as fusion proteins in *E. coli*, which can limit yields after isolation and purification. We recently reported that peptide degradation may be prevented by production of a “sandwiched” SUMO-peptide-intein (SPI) fusion protein, which protects the target peptide sequence from truncation and improves yield. This initial system required cloning with two commercially available vectors. It used an N-terminal polyhistidine tagged small ubiquitin-like modifier (SUMO) protein and a C-terminal engineered *Mycobacterium xenopii* DNA Gyrase A intein with an inserted chitin binding domain (CBD) to create “sandwiched” fusion proteins of the form: His_6_-SUMO-peptide-intein-CBD. However, the major drawback of this previously reported fusion protein “sandwich” approach is the increased time and number of steps required to complete the cloning and isolation procedures, relative to the simple procedures to produce recombinant peptides in *E. coli* from a single (non-“sandwiched”) fusion protein system.

**Results:**

In this work we generate the plasmid pSPIH6, which improves upon the previous system by encoding both the SUMO and intein proteins and allows facile construction of a SPI protein in a single cloning step. Additionally, the *Mxe* GyrA intein encoded in pSPIH6 contains a C-terminal polyhistidine tag, resulting in SPI fusion proteins of the form: His_6_-SUMO-peptide-intein-CBD-His_6_. The dual polyhistidine tags greatly simplify isolation procedures compared to the original SPI system, which we have here demonstrated with two linear bacteriocin peptides: leucocin A and lactococcin A. The yields obtained for both peptides after purification were also improved compared to the previous SPI system as a result of this streamlined protocol.

**Conclusions:**

This modified SPI system and its simplified cloning and purification procedures described here may be generally useful as a heterologous *E. coli* expression system to obtain pure peptides in high yield, especially when degradation of the target peptide is an issue.

**Supplementary Information:**

The online version contains supplementary material available at 10.1186/s12896-023-00779-5.

## Background

Peptides are a class of biomolecules with established importance in industries including agriculture [1], pharmaceuticals [2], food [3], and cosmetics [4]. While many peptides can be produced using solid phase peptide synthesis [5], ribosomally synthesized peptides may be produced for research or industrial purposes using bacterial fermentation, as this method is relatively economical, green, and scalable [6].

Some peptide natural products may be readily isolated from the original producer organism, while others may be expressed by the host in only tiny amounts or under conditions that cannot be easily replicated in the laboratory [7]. In these cases, heterologous expression in *Escherichia coli* may offer a solution. Recombinant peptide production can be achieved by construction of a fusion protein that contains an affinity tag to aid in isolation, as well as a protease cleavage site to allow for separation of the peptide from the fusion protein during isolation and purification [8].

Common fusion proteins used in heterologous expression include glutathione *S-*transferase, small ubiquitin-like modifier (SUMO), intein proteins, thioredoxin, and maltose binding protein [9]. SUMO, inteins, and thioredoxin require an additional affinity tag to be included in the construct for easy isolation using affinity purification. A polyhistidine sequence is usually the affinity tag of choice, as immobilized metal ion chromatography using a resin such as a nickel nitrilotriacetic acid (Ni-NTA) has a very high binding capacity [10, 11]. Other possible affinity tags include a chitin-binding domain (CBD) or maltose binding protein. Possible proteases and associated recognition sequences that can be used in fusion proteins include tobacco etch virus protease, factor Xa protease, enterokinase, and thrombin [12]. However, use of protease recognition sequences may be nonspecific or leave behind extra residues on the peptide of interest after cleavage. Importantly, use of an N-terminal His_6_-SUMO fusion tag, followed by addition of its corresponding SUMO protease during the isolation process will leave behind no extra residues on the peptide of interest [13]. Use of a C-terminal intein fusion protein will also produce a scarless cleavage site on the peptide of interest [14].

Combinations of multiple fusion proteins and/or affinity tags have also been reported. This can be in the form of simple addition of a polyhistidine sequence to other affinity tags, which is useful because Ni-NTA agarose has a higher binding capacity than most other affinity chromatography resins [15–22]. Dual or tandem fusion protein tags have been used to increase solubility of a target protein or offer unique purification strategies [23–29]. Still, other fusion proteins have been constructed with one or more flanking fusion tags on both the N- and C- termini of a protein of interest, thereby creating a fusion protein “sandwich” around a target protein [30–33]. Notably, when the “sandwiching” fusion proteins surrounding a target protein sequence are an N-terminal SUMO protein and a C-terminal intein, cleavage of each fusion protein will leave behind no extra residues on the target protein sequence [34, 35].

We recently reported the use of this dual SUMO and intein “sandwiched” expression system to protect peptides from degradation during heterologous expression in *E. coli* [36]. In that work, we initially expressed four different peptides as simple SUMO fusion proteins, and found that the final yield of each purified peptide was very low due to truncations in the peptide sequences incurred during expression. We then showed that expression of each of these peptides as “sandwiched” SUMO-peptide-intein (SPI) fusion proteins prevented degradation and significantly improved final yields of the four peptides. However, one of the main disadvantages of using this SPI system is the significant increase in time needed for the cloning and isolation procedures, compared to that needed to clone and isolate a non-“sandwiched” fusion protein [37].

We here describe an updated SPI fusion protein system with simplified cloning and isolation procedures. The plasmid pSPIH6 was constructed, and this vector enables single step cloning construction of a SPI fusion protein with NEBuilder® HiFi DNA Assembly, which is based on the principles of Gibson assembly. The constructed SPI protein leaves behind no extra residues on the target peptide sequence after removal of the two fusion protein tags. The intein encoded in pSPIH6 (*Mxe* GyrA intein) includes an additional C-terminal polyhistidine tag to allow for a simplified purification process for any target peptide. The described isolation procedure has fewer steps, does not require chitin resin, and produces SPI fusion proteins of the form: His_6_-SUMO-peptide-intein-CBD-His_6_. Protein precipitation during intein cleavage was also addressed using a screen for buffer additives to prevent aggregation and insolubility. We used this simplified SPI system to clone, express, and purify the linear bacteriocin peptides leucocin A and lactococcin A (Table 1), which were previously shown to be truncated when expressed as simple SUMO fusion proteins [36]. The peptides were each purified and obtained in increased yield compared to the originally reported SPI system, likely due to the lower number of purification steps required. This updated and simplified SPI fusion system may be generally useful as a first choice of expression system for peptides and proteins (not just those that undergo degradation) as its cloning and isolation procedures are nearly the same number of steps as those for single fusion proteins.

**Table 1:** Comparison of peptide sequences used in this study

Peptide	Amino acid sequence	Molecular weight (g/mol)	Number of residues
LeuA	KYYGNGVHCTKSGCSVNWGEAFSAGVHRLANGGNGFW	3929.8	37
LcnA	KLTFIQSTAAGDLYYNTNTHKYVYQQTQNAFGAAANTIVNGWMGGAAGGFGLHH	5774.8	54

## Results

**Fig. 1:**
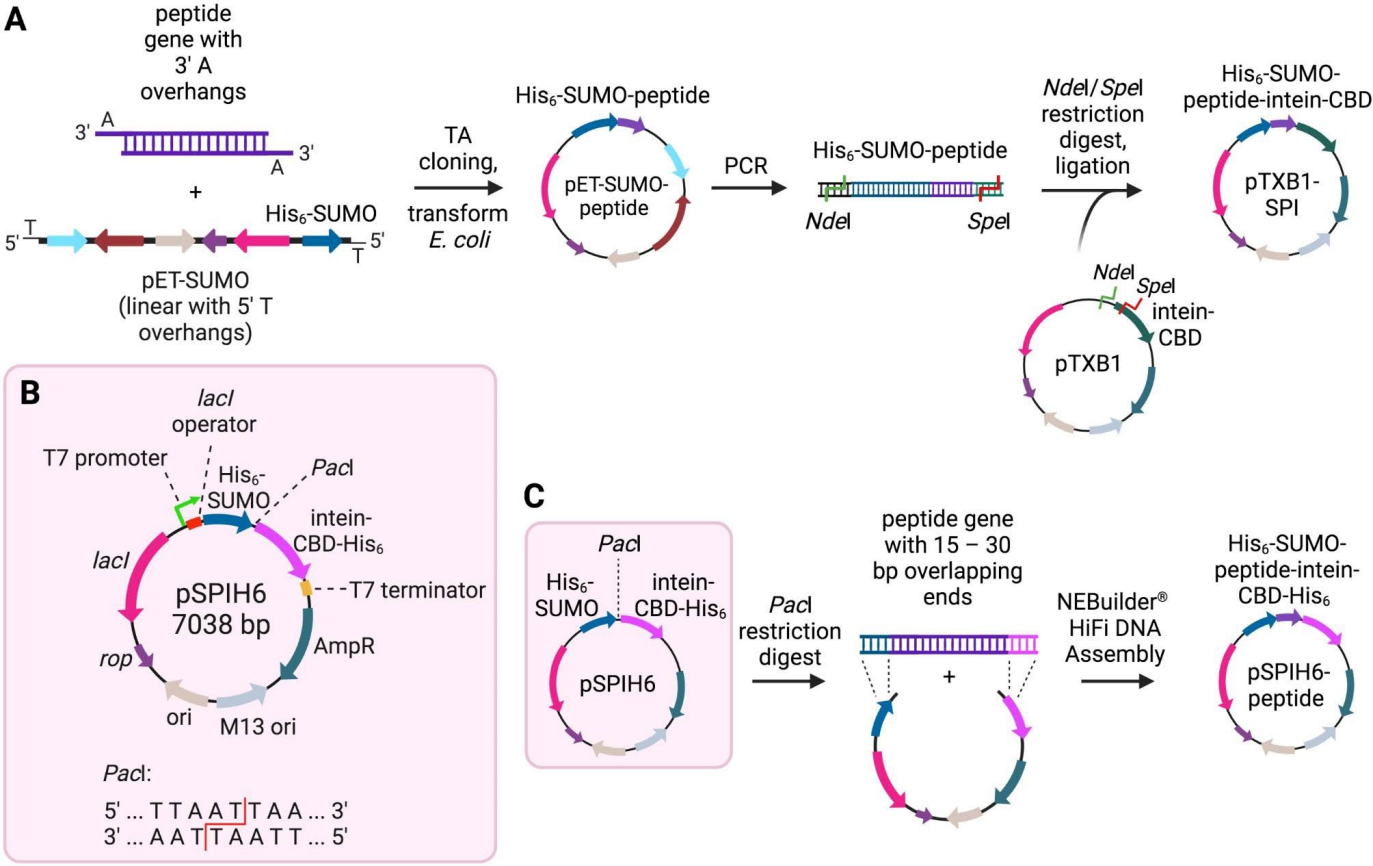
Comparison of previously reported SPI fusion protein cloning protocol with the new protocol outlined in this work. **(A)** Previously reported cloning protocol to construct a SPI fusion protein from pET-SUMO and pTXB1 vectors (Lamer et al., 2022a). The desired peptide gene is amplified with PCR and inserted into the linearized pET-SUMO vector with TA cloning. The His_6_-SUMO-peptide gene is then amplified with PCR, and flanking 5’ *Nde*I and 3’ *Sap*I restriction sites are inserted with appropriate DNA primer design. The PCR product and pTXB1 plasmid are both doubly digested with *Nde*I and *Sap*I, and then ligated together to construct the pTXB1-SPI plasmid with the target peptide gene inserted seamlessly between His_6_-SUMO and intein-CBD genes. **(B)** Vector map for pSPIH6, based on the pTXB1 backbone. The His_6_-SUMO and intein-CBD-His_6_ genes are separated by a *Pac*I restriction site to allow insertion of a target peptide gene. An ampicillin resistance gene acts as a selective marker. Expression of a SPI fusion protein is under the control of a T7 promoter, and can be induced with the addition of IPTG to the culture media. **(C)** Simplified cloning protocol used in this work to construct a SPI fusion protein from pSPIH6. The plasmid is first digested with *Pac*I. The target peptide gene is amplified with PCR, and DNA primers are designed to create 15–30 bp overlaps on the 5’ and 3’ ends of the target gene sequence. These 5’ and 3’ overlapping regions are identical to the 3’ end of the His_6_-SUMO gene and the 5’ end of the intein-CBD-His_6_ genes, respectively, to allow for NEBuilder® HiFi DNA Assembly with the linearized pSPIH6 vector. The bases of the *Pac*I restriction site are removed with 5’ and 3’ exonuclease activity of the DNA Polymerase included in the NEBuilder® kit to create a seamless SPI gene in the newly constructed pSPIH6-peptide plasmid

**Fig. 2:**
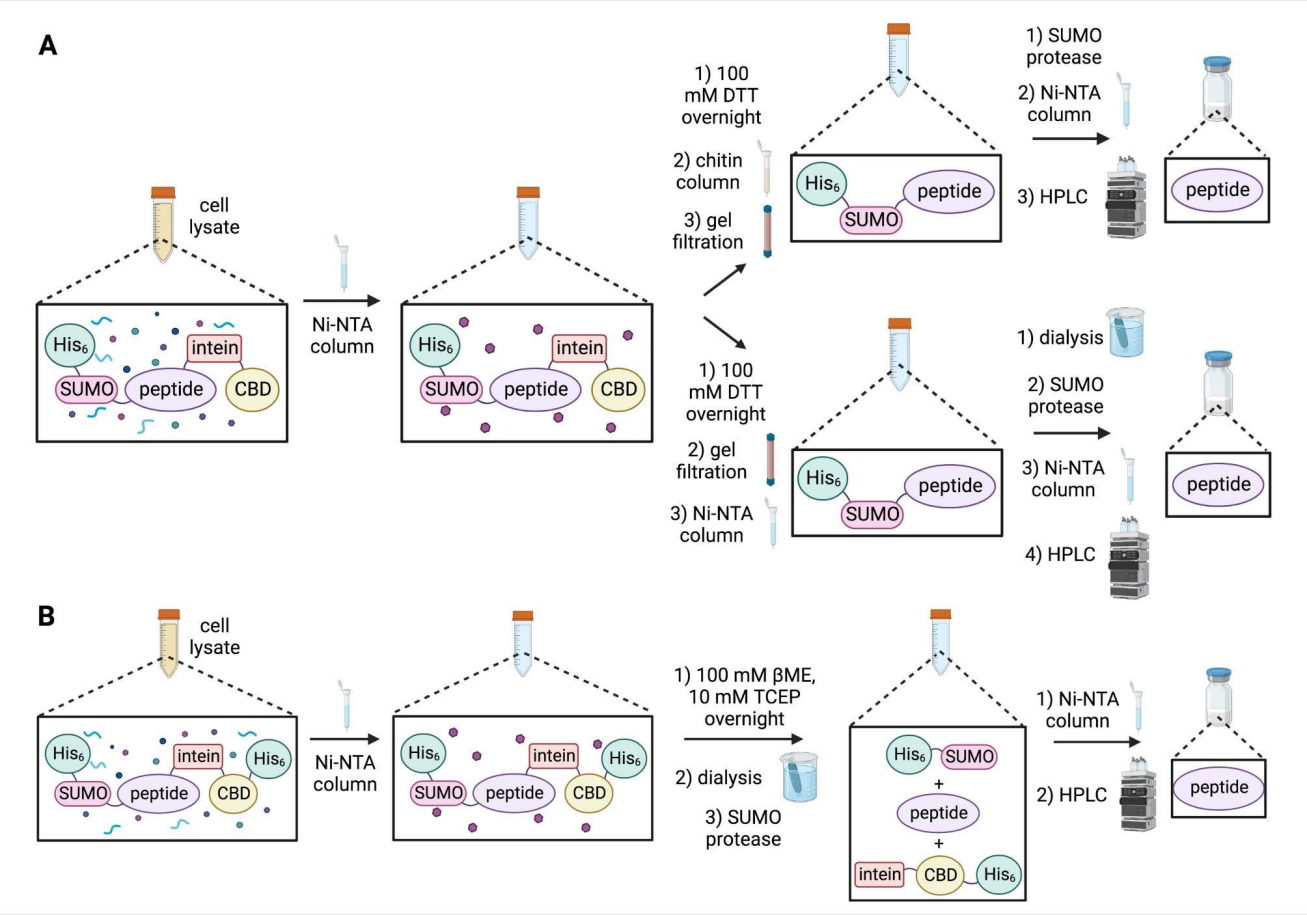
Comparison of previously reported SPI fusion protein isolation protocols with the new protocol outlined in this work. **(A)** Previously reported isolation protocols to obtain pure peptide from a SPI fusion protein (Lamer et al., 2022a). Clarified cell lysate is passed over a Ni-NTA column, and the SPI fusion protein is eluted with imidazole (purple hexagons). The intein is then cleaved via incubation in 100 mM DTT overnight, and then removed from the sample with a chitin column (top arrow). DTT and imidazole are removed with gel filtration chromatography prior to addition of the SUMO protease. The cleaved His_6_-SUMO tag and His-tagged SUMO protease are removed with a second Ni-NTA column, and the peptide in the column flow through is finally purified with HPLC. Alternatively, if the use of chitin resin is undesirable (bottom arrow), after intein cleavage the buffer is exchanged with gel filtration to remove imidazole and DTT, and the His_6_-SUMO-peptide protein is separated from the intein using a second Ni-NTA column. Imidazole is then removed from the eluted protein sample with dialysis, and then cleavage by the SUMO protease, a third Ni-NTA column, and HPLC are conducted to obtain pure peptide. **(B)** Simplified isolation protocol used in this work to obtain pure peptide from a SPI fusion protein. The SPI fusion protein is isolated from the clarified cell lysate using a Ni-NTA column, and then the intein is cleaved from the eluted SPI protein by addition of 100 mM βME. Reducing agents and imidazole are then removed with dialysis, and addition of the SUMO protease cleaves the His_6_-SUMO tag from the peptide. The peptide is then isolated using a second Ni-NTA column, and finally purified with HPLC.

While it has been previously shown that the SPI system can significantly improve yields of purified peptides that undergo degradation during heterologous expression in *E. coli*, a major drawback of using the originally reported SPI system is the high number of steps and time required for construction of the expression vector (Fig. 1A) and for the purification process (Fig. 2A). To simplify the SPI cloning process, a single *E. coli* expression vector containing both the His_6_-SUMO and intein-CBD-His_6_ genes was constructed based on the pTXB1 vector backbone (Supplementary Fig. 1). The intein-CBD gene was PCR amplified from pTXB1 using a forward primer that included the upstream *Sap*I restriction site, and a reverse primer that included an additional C-terminal polyhistidine tag DNA prior to the stop codon, and the *Pst*I restriction site immediately following the stop codon. Digestion of pTXB1 and the PCR product with *Pst*I and *Sap*I followed by ligation generated the vector pTXIH6, which is identical to pTXB1 except that the encoded intein contains an extra C-terminal polyhistidine tag. Next, the His_6_-SUMO gene encoded in a previously constructed pET-SUMO-LeuA vector was amplified using primers that included specific complementary tail sequences for insertion into a *Nde*I and *Sap*I digested pTXIH6 vector using NEBuilder® HiFi DNA Assembly. The reverse primer also included a *Pac*I restriction site directly following the final glycine codon of the SUMO gene. Insertion of the His_6_-SUMO gene into *Nde*I and *Sap*I digested pTXIH6 yielded the final construct pSPIH6, which encodes a His_6_-SUMO gene followed by an intein-CBD-His_6_ gene, with these two fusion protein coding sequences being separated by a unique *Pac*I restriction site (Fig. 1B).

The *Pac*I site in pSPIH6 allows for single step, seamless insertion of any target peptide gene between the last glycine codon of the His_6_-SUMO gene and the first cysteine codon (catalytic cysteine) of the intein-CBD-His_6_ gene. Two bacteriocin peptide genes, leucocin A (LeuA) and lactococcin A (LcnA), were each cloned into pSPHI6 (Fig. 1C). This was achieved by PCR amplification of the target peptide gene using primers that include complementary tail sequences to pSPIH6 on either side of the *Pac*I restriction site. NEBuilder® HiFi DNA Assembly uses a DNA polymerase with both 5’ and 3’ exonuclease activity, allowing for abolition of the *Pac*I restriction site and seamless, in-frame insertion of the peptide gene between the two flanking fusion protein genes.

Both LcnA and LeuA SPI fusion proteins were overexpressed in *E. coli* BL21(DE3) using isopropyl β-D-1-thiogalactopyranoside (IPTG) to induce SPI gene expression under the control of a T7 promoter. LcnA was induced at a lower temperature (15 °C overnight) because the final residue preceding the intein’s catalytic cysteine is a histidine, which can lead to premature intein hydrolysis during expression at 37 °C, as previously described [36] and predicted [38].

**Fig. 3:**
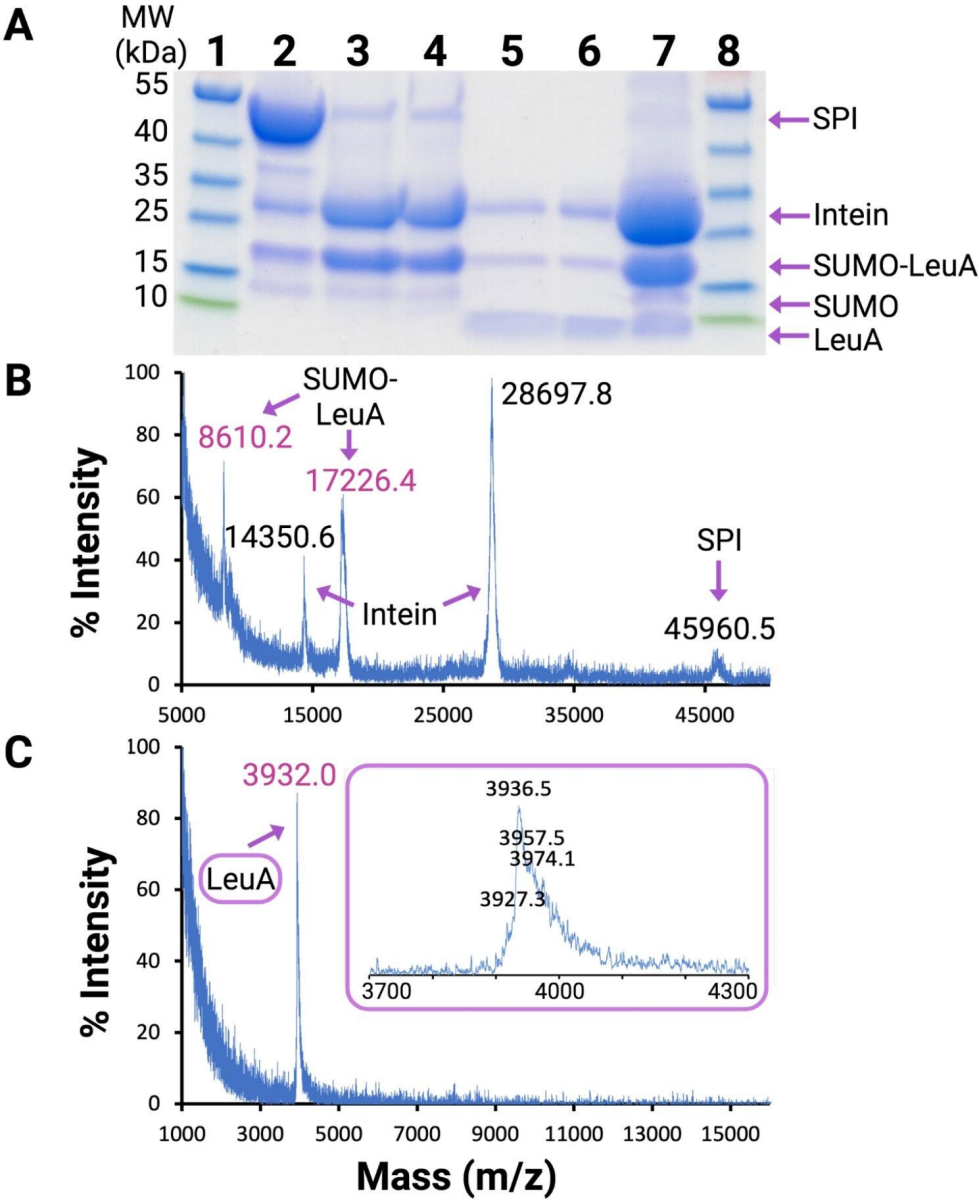
Isolation and purification of LeuA as a SPI fusion protein. **(A)** SDS-PAGE results over the course of LeuA purification. MW = molecular weight in kilodaltons; 1 = molecular weight protein ladder; 2 = pooled fractions of first Ni-NTA column; 3 = after overnight incubation in 100 mM βME; 4 = after dialysis; 5 = second Ni-NTA column flow through; 6 = second Ni-NTA column 5 mM imidazole wash; 7 = second Ni-NTA column 500 mM imidazole wash; 8 = molecular weight protein ladder. **(B)** MALDI-TOF MS after intein cleavage. **(C)** MALDI-TOF MS of pure LeuA after HPLC. The inlaid purple box shows an expansion of the LeuA mass peak

**Fig. 4:**
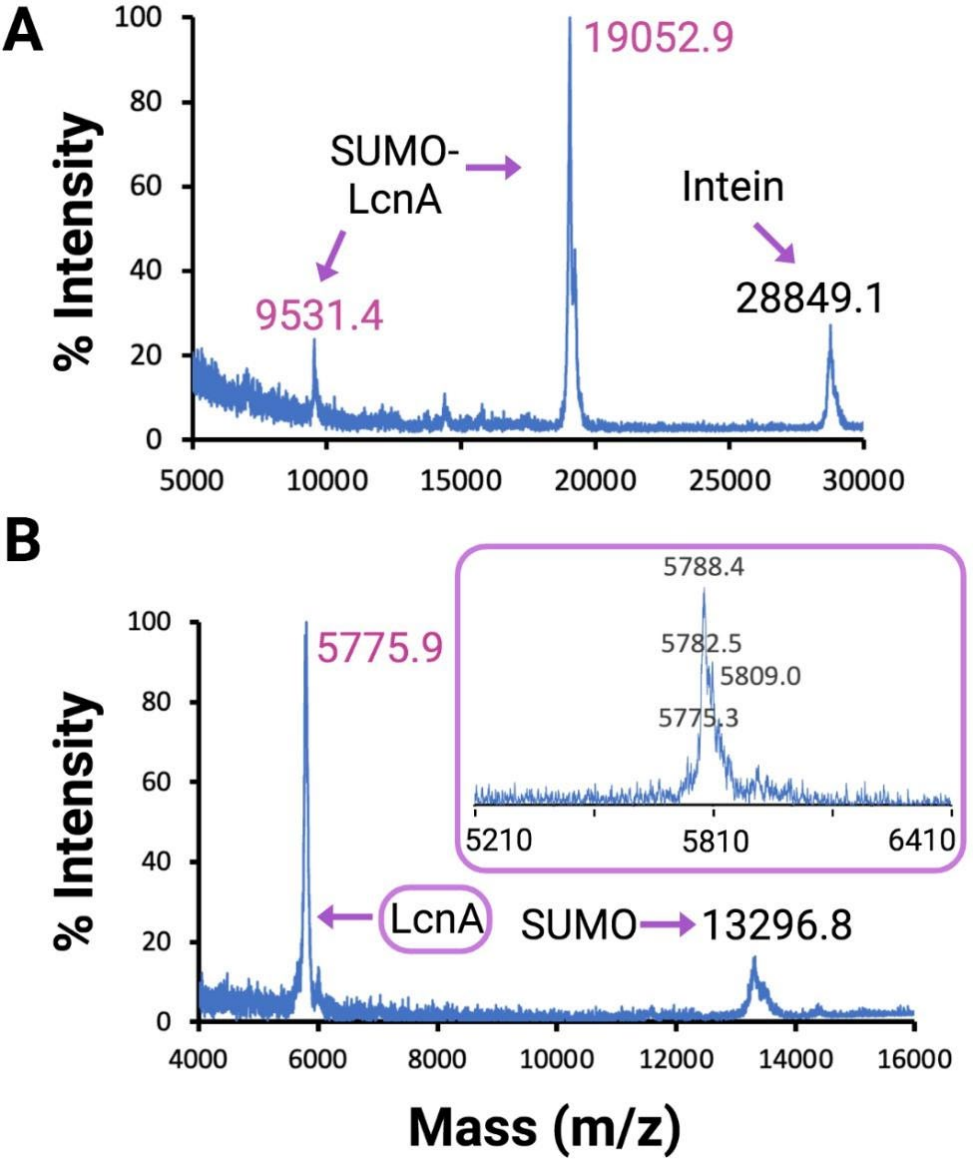
Isolation and purification of LcnA as a SPI fusion protein. **(A)** MALDI-TOF MS of SUMO-LcnA after intein cleavage. **(b)** MALDI-TOF MS of LcnA after SUMO cleavage. The inlaid purple box shows an expansion of the LcnA mass peak

In addition to simplifying the cloning process for construction of SPI fusion proteins, the isolation and purification procedures were also simplified as a result of the extra C-terminal polyhistidine tag on the intein-CBD fusion protein (Fig. 2B). One issue encountered with both the LeuA and LcnA isolation processes was protein precipitation during the intein cleavage step. To address this issue, a screen of various buffer additives was performed on the LcnA SPI fusion protein to identify intein cleavage conditions that prevented precipitation (Supplementary Fig. 2A). Samples were gently rotated for 72 h at 4 °C, centrifuged to pellet any insoluble protein, and then SDS-PAGE was performed on the soluble fraction. Interestingly, the conditions that prevented protein precipitation were with no thiol added (no intein cleavage and SPI fusion protein kept intact), addition of 1% (v/v) of the non-ionic detergent IGEPAL® CA-630, and addition of 500 mM arginine hydrochloride. All other conditions displayed visual precipitation and SDS-PAGE of the soluble fraction after centrifugation showed disappearance of the SUMO-LcnA band. The identity of the precipitate was found to be SUMO-LcnA by SDS-PAGE of the resuspended insoluble precipitate (Supplementary Fig. 2B). An additional screen of lower concentrations of arginine hydrochloride and IGEPAL® CA-630, as well as combinations of these two additives showed that 250 mM arginine hydrochloride and 0.01% IGEPAL® CA-630 together were able to prevent protein precipitation during intein cleavage (Supplementary Fig. 2C). LeuA and LcnA were both finally purified under these conditions (Figs. 3 and 4), resulting in improved pure peptide yields compared to the originally reported SPI system (Table 2), and were active in an antimicrobial activity assay against *Lactococcus lactis* subsp. *lactis* IL1403 (data not shown).

**Table 2:** Comparison of yields of peptides after purification with SUMO and SPI systems

Peptide	Purified peptide yield as SUMO fusion protein (mg)*	Purified peptide yield from original SPI system (mg)*	Purified peptide yield from simplified SPI system (mg)
LeuA	0.1	1.7	1.9
LcnA	< 0.1 mg	0.7	3.0

*Values previously reported [36]

## Discussion

Degradation of peptides heterologously expressed as fusion proteins in *E. coli* can be a major challenge. Optimization of expression conditions to suppress or minimize degradation is extremely time consuming, and it is difficult to predict whether it will improve results. We previously showed that expression of a “sandwiched” fusion protein of the form His_6_-SUMO-peptide-intein-CBD in *E. coli* can protect peptides against degradation and result in improved yields [36]. Here, the SPI system was optimized by creating a single expression vector, pSPIH6 (Addgene plasmid #190676), that encodes both fusion proteins and allows for construction of a SPI fusion protein in a single cloning step. This universal plasmid is a significantly cheaper and faster option to construct a SPI fusion protein than beginning from commercially available pET-SUMO and pTXB1 vectors. pSPIH6 allows for insertion of any peptide gene sequence, and results in scarless cleavage sites and no extra residues left behind on the target peptide sequence after purification. This is particularly important for peptides, as any additional residues may impact structure and function of these small proteins.

The addition of a C-terminal polyhistidine tag to the *Mxe* GyrA intein has been reported previously [39] and allows the use of Ni-NTA affinity chromatography during the isolation process, rather than chitin affinity columns. Immobilized metal ion affinity chromatography is preferential because of the resin’s higher binding capacity compared to other types of affinity chromatography [11]. In this work, addition of the extra C-terminal polyhistidine tag on the SPI construct (His_6_-SUMO-peptide-intein-CBD-His_6_) also greatly simplified the isolation procedure, and the fewer number of steps led to an improved yield compared to the originally reported SPI system for both LeuA and LcnA. Several conditions in the isolation process were also modified based on a literature method [39]. The Tris lysis buffer was changed to a phosphate buffer to prevent reaction of Tris with thioester-containing proteins [40]. Intein cleavage conditions were modified from 100 mM D,L-dithiothreitol (DTT) to 100 mM 2-mercaptoethanol (βME) with 10 mM tris(2-carboxyethyl)phosphine (TCEP) because intein cleavage was faster with βME than DTT (data not shown). This may be due to increased stability of βME during the overnight cleavage reaction compared to DTT. Ethylenediaminetetraacetic acid (EDTA) was also added to the intein cleavage reaction to chelate any Ni^2+^ eluted from the Ni-NTA column and prevent oxidation of βME.

Intein cleavage conditions required optimization and screening of buffer additives to prevent precipitation of the SUMO-peptide fusion proteins [41]. Addition of 250 mM arginine and 0.01% (v/v) IGEPAL® CA-630 were found to prevent precipitation. Lower concentrations of arginine without detergent present did not prevent precipitation (Supplementary Fig. 2A). The subsequent dialysis step is extremely important to remove arginine, EDTA, βME, and TCEP, which each may interfere with the SUMO protease and/or subsequent Ni-NTA affinity chromatography. Despite the low concentration, 0.01% (v/v) IGEPAL® CA-630 is still above the critical micellar concentration of this detergent (0.083 mM), which may be why it is still sufficient to suppress protein precipitation. As dialysis does not remove the micellar detergent, it was important to use as small a concentration as possible to prevent issues with high performance liquid chromatography (HPLC) purification.

While it cannot be predicted if the combination of arginine and IGEPAL® CA-630 will work to keep all SUMO-peptide fusion proteins soluble during intein cleavage of SPI fusion proteins, a general buffer additive screening method was implemented here that could be easily repeated for other SPI fusion proteins. It is possible that in other SPI constructs the SUMO-peptide fusion protein may remain fully soluble during intein cleavage and buffer additives are not required. In these cases, it would be better to perform intein cleavage without detergent present to simplify HPLC purification.

There are several considerations when choosing to use the SPI system for peptide expression and purification. For efficient SUMO cleavage by the SUMO protease, the first residue of the peptide following the diglycine cleavage site of the SUMO tag cannot be a proline. Likewise, the *Mxe* GyrA intein used in this SPI system has preferences for certain residues preceding the catalytic cysteine. Most residues are tolerated, though some such as histidine are predicted to lead to premature intein hydrolysis [38]. LcnA is one such peptide sequence that terminates in a histidine residue, but it is possible to minimize premature intein cleavage with lower temperature induction conditions. Still, other residues are predicted to lead to slower intein cleavage, though these are predictions and must be tested on a case by case basis. It may also be possible to swap the intein included in the SPI system to other inteins with improved cleavage parameters, solubility, or residue preferences. Finally, the use of a thiol reducing agent to cleave the intein from the SPI fusion protein reduces any disulfide bonds in the peptide, and so a refolding step may be required to reform native disulfides.

While this work addresses the degradation of peptides during *E. coli* heterologous expression by protecting the peptide sequence between cleavable SUMO and intein proteins, recent reports have shown the use of similar traceless SUMO and intein fusion protein “sandwiches” for other purposes. Osunsade et al. reported a SUMO and intein “sandwich” to successfully produce C-terminal domains of histone proteins, which are highly positively charged and are often degraded during heterologous expression [35]. Raducanu et al. developed an expression system with SUMO and intein sequences flanking a protein of interest for scarless cleavage, but with additional thioredoxin proteins fused to both the SUMO and intein fusion proteins to improve solubility [34]. The “sandwiching” expression strategy using an N-terminal SUMO protein and a C-terminal intein, which each allow for traceless removal of the fusion protein and no extra residues left behind on a target protein or peptide sequence, may become a general fusion protein expression strategy and be especially useful to produce proteins or peptides that are prone to degradation.

## Conclusions

While previously reported SPI expression systems required time-consuming cloning and isolation procedures and would not be recommended for use unless degradation of the target peptide sequence was occurring, the simplicity of the updated SPI system described here may make it an attractive first choice expression system for many target peptides or proteins, even when degradation is not an issue.

## Materials and methods

### General method information

Commercially available chemical and biological reagents were purchased from Sigma-Aldrich Canada, Bio-Rad Laboratories, Chem-Impex International Inc., or Thermo Fisher Scientific, and used without further purification unless otherwise stated. HPLC grade acetonitrile was used without further purification. Deionized water was obtained from a Milli-Q reagent water filtration system (Millipore Co., Milford, MA). Semi-preparative scale HPLC was performed on an Agilent chromatograph equipped with a 1260 Infinity II quaternary pump, a 1260 Infinity II diode array detector, a 1260 Infinity II fraction collector, and a 1260 Infinity II manual injector fitted with a 500 µL sample loop. The column used for HPLC was a Vydac 219TP Diphenyl, 5 µ, 10 × 250 mm. HPLC solvents were filtered through a Millipore filtration system under vacuum prior to use. Mass spectra were recorded on a Perspective Biosystems Voyager™ Elite matrix-assisted laser desorption ionization time of flight mass spectrometry (MALDI-TOF MS) using sinapinic acid as a matrix, and data was analyzed with Agilent Mass Hunter Qualitative Analysis software Package (version B.03.01 SP3). The molecular weight marker used in SDS-PAGE was PageRuler Prestained Protein Ladder, 10–180 kDa (Thermo Scientific). *E. coli* plasmids were purified using GeneJET Plasmid Miniprep Kit (Thermo Scientific), and linearized plasmids and PCR products were purified using GeneJET PCR Purification Kit (Thermo Scientific). PCR reactions were completed using Phusion® High-Fidelity DNA Polymerase (New England BioLabs), and all restriction enzymes were obtained from New England BioLabs and reactions were performed according to manufacturer recommendations. Supplementary methods including DNA primer sequences, DNA and protein sequences, HPLC chromatograms, and MALDI-TOF MS spectra can be found in the supplementary information file.

### Construction of pTXIH6 and pSPIH6 plasmids

From pTXB1 the *Mxe* GyrA intein was amplified using primers *Int-F* and *Int-R*, which added a C-terminal polyhistidine (His_6_) tag to the protein sequence, as well as 5’ *Sap*I and 3’ *Pst*I restriction sites in the PCR product. The PCR product was purified and then the pTXB1 vector (New England BioLabs) and the PCR product were each doubly digested with *Sap*I and *Pst*I. The linear DNA products were each purified, and then the digested PCR product was inserted into the linearized pTXB1 plasmid using T4 DNA Ligase. NEB® 10-beta Competent *E. coli* were then transformed with the ligation mixture, and transformed cells were plated out on Difco™ Luria Broth (LB) media plates (10 g of tryptone, 5 g of yeast extract, 10 g of NaCl) with ampicillin added (100 µg/mL) as selective pressure. Plates were grown overnight at 37 °C, and then colony PCR was performed with primers *Col-Int-F* and *Col-Int-R* to screen for transformants containing the His-tagged intein insert. Potential positive transformant pTXIH6 colonies were then grown in 5 mL of LB media overnight, purified, and sequenced with Sanger sequencing using primers *Int-seq-F-1*, *Int-seq-R-1*, *Int-seq-F-2*, and *Int-seq-R-2*. Cells containing the correct His-tagged intein insert (pTXIH6) were stored as 20% glycerol stocks at -80 °C.

From pET-SUMO-LeuA, the His_6_-SUMO gene was amplified using primers *SUMO-F* and *SUMO-R*, which included specific tail sequences for insertion into *Nde*I and *Sap*I digested pTXIH6 using NEBuilder® HiFi DNA Assembly. The PCR product was purified, and then pTXIH6 was digested with *Nde*I and *Sap*I, followed by purification of the linearized plasmid. NEBuilder® HiFi DNA Assembly Master Mix was then used to insert the SUMO PCR product into the linearized pTXIH6 vector. NEB® 10-beta Competent *E. coli* were then transformed with the product mixture, and transformed cells were plated on LB media with ampicillin added (100 µg/mL) as selective pressure. Plates were grown overnight at 37 °C, and then colony PCR was performed with primers *SUMO-Col-F* and *SUMO-Col-R* to screen for transformants containing the SUMO gene. Potential positive transformant pSPIH6 colonies were then grown in 5 mL of LB media overnight, purified, and sequenced with Sanger sequencing using primers *SUMO-Col-F* and *SUMO-Col-R*. Cells containing the correct insert (pSPIH6) were stored as 20% glycerol stocks at -80 °C.

### General method for cloning peptide genes into pSPIH6

pSPIH6 is available through Addgene (plasmid #190,676). LeuA and LcnA peptide genes were amplified from previously constructed pTXB1-SPI-LeuA and pTXB1-SPI-LcnA vectors [36] using primers that included specific tail sequences for insertion into a *Pac*I digested pSPIH6 vector using NEBuilder® HiFi DNA Assembly (Supplementary Table 1). The primers used were *LeuA-G-F* and *LeuA-G-R* for LeuA, and *LcnA-G-F* and *LcnA-G-R* for LcnA. pSPIH6 was digested with *Pac*I and the linearized vector and peptide gene PCR products were purified. PCR products were inserted into the *Pac*I digested pSPIH6 using NEBuilder® HiFi DNA Assembly Master Mix. NEB® 10-beta Competent *E. coli* were then transformed with the product mixture, and transformed cells were plated on LB media with ampicillin added (100 µg/mL) as selective pressure. Plates were grown overnight at 37 °C, and then colony PCR was performed with universal primers *SPI-col-F* and *SPI-col-R* to screen for transformants containing the desired peptide gene inserted into pSPIH6. Potential positive transformant colonies were then grown in 5 mL of LB media overnight, purified, and sequenced with Sanger sequencing using universal primers *TL-pTXB1-F* and *SUMO-Col-R*. Cells containing the correct insert (pSPIH6-LeuA or pSPIH6-LcnA) were stored as 20% glycerol stocks at -80 °C, and then *E. coli* BL21(DE3) cells were transformed with the desired pSPIH6-peptide plasmids for protein production.

### General SPI protein expression and isolation procedures

Frozen glycerol stocks of *E. coli* BL21(DE3) cells transformed with the appropriate pSPIH6-peptide vector were inoculated into 50 mL of sterile LB media with ampicillin added (100 µg/mL) as selective pressure. The cells were grown overnight at 37 °C with shaking at 225 rpm. The next day, 20 mL of the overnight culture was added to 500 mL of sterile LB media with ampicillin added, and cells were grown to an optical density (OD_600_) of 0.8 at 37 °C. Protein expression was then induced by addition of IPTG at final concentrations of 0.1 mM for induction at 15 °C overnight (~ 20 h), or 0.5 mM for induction at 37 °C for 4 h. If cells were to be induced at 15 °C, the flask was first cooled in an ice bath before adding IPTG. Cultures were shaken at 225 rpm during induction. Cells were then harvested by centrifugation (5,000 × *g*, 10 min, 4 °C) and the pellets were stored at -80 °C. In this study, cells expressing the LeuA SPI fusion protein were induced at 37 °C for 4 h, while cells expressing the LcnA SPI fusion protein were induced at 15 °C overnight.

The frozen cell pellet was resuspended (30 mL buffer/500 mL culture media) evenly in ice cold lysis buffer (50 mM NaH_2_PO_4_, 300 mM NaCl, 5 mM imidazole, pH 8.0) by vortexing. Cells were lysed by sonication while kept on ice (20% amplitude, constant pulse, 15 s on and 15 s off, 6 min total). DNase I (Thermo Scientific, 1 U) was added and the lysate kept on ice for 15 min with occasional inversion. The cellular debris was removed by centrifugation (20,000 × *g*, 30 min, 4 °C), and then pre-washed Ni-NTA resin (Qiagen) was added to the clarified supernatant (3 mL of resin/500 mL culture media) and the mixture was gently shaken for 1 h at 4 °C. This was then loaded onto a fritted column and the flow through collected at 4 °C. The resin was washed with 25 mL of lysis buffer containing 20 mM imidazole, and then the fusion protein was eluted in 5 mL fractions by sequential addition of elution buffer (50 mM NaH_2_PO_4_, 300 mM NaCl, pH 8.5) containing 40, 60, 80, 100, 200, and then 500 mM imidazole. Eluted fractions were analyzed by sodium dodecyl sulfate polyacrylamide gel electrophoresis (SDS-PAGE) and/or MALDI-TOF MS and the samples containing the protein of interest were pooled together. The pooled fractions had 5 mM EDTA, 100 mM βME, 10 mM TCEP, 250 mM arginine hydrochloride, and 0.01% (v/v) IGEPAL® CA-630 added and were adjusted to pH 8.5 if needed. The sample was gently rotated overnight at 4 °C to cleave the intein. Intein cleavage completion was assessed with SDS-PAGE and/or MALDI-TOF MS. The sample was then dialyzed (molecular weight cut off 6,000–8,000 kDa) in 1 L of SUMO buffer (50 mM NaH_2_PO_4_, 150 mM NaCl, 1 mM DTT, pH 8) at 4 °C for 4 h with two buffer changes to remove imidazole, reducing agents, arginine, and EDTA. The sample was then removed to a glass vial and His-tagged SUMO protease was added (McLab, South San Francisco, CA, 1000 units/500 mL culture media). The sample was gently rotated at 4 °C for 2–4 h, and cleavage progress was assessed with MALDI-TOF MS. The His_6_-SUMO tag, His-tagged SUMO protease, and His-tagged intein were then removed with a second Ni-NTA column at 4 °C. The flow through and wash fractions were pooled and then the crude peptide was lyophilized and redissolved in an acetonitrile/water mixture with 0.1% trifluoroacetic acid. The peptide was further purified using semi-preparative HPLC and then lyophilized to obtain a final yield of purified peptide per L of culture media.

### HPLC purification methods

HPLC purification was completed with aqueous 0.1% trifluoroacetic acid (TFA) (solvent A) and 0.1% TFA in acetonitrile (solvent B) as eluents. Semi-prep purification and reinjection analyses were conducted at a flow rate of 5 mL/min with wavelength monitoring at 220, 254, and 280 nm. Peptide purity was confirmed by both MALDI-TOF MS and HPLC reinjection of a sample of the pooled peptide-containing fractions. LeuA was stirred overnight in 1 mM TCEP prior to HPLC injection to reduce disulfide bonds. LeuA MS (MALDI-TOF) Calculated for C_174_H_248_N_52_O_50_S_2_ 3929.8, found 3930.9 (M + H)^+^. LcnA MS (MALDI-TOF) Calculated for C_260_H_380_N_72_O_77_S_1_ 5774.8, found 5775.9 (M + H)^+^. **HPLC Method 1 (LeuA purification, peptide elutes at 39% B)**: 0 − 8 min, 15% B; 8 − 20 min, ramp 15 − 60% B; 20–20.5 min, ramp 60 − 100% B; 20.5 − 24 min, 100% B. **HPLC Method 2 (LcnA purification, peptide eluted at 42% B)**: 0 − 4 min, 20% B; 4 − 13 min, ramp 20 − 46% B; 13–13.5 min, ramp 46 − 100% B; 13.5 − 20 min, 100% B.

### Screen of buffer additives to prevent protein precipitation during intein cleavage

This screen was adapted based on a literature procedure [41]. SPI fusion proteins were initially expressed and isolated with the first Ni-NTA column as described above. The Ni-NTA column was eluted with 30 mL of lysis buffer (50 mM NaH_2_PO_4_, 300 mM NaCl, pH 8.0) with 150 mM imidazole added. 1 mL aliquots were portioned into microcentrifuge tubes at 4 °C. Additives were added as dry powders or neat liquids, and pH was readjusted to 8.5 after addition if needed. The samples were gently shaken for 72 h at 4 °C, and then centrifuged (2 min, 13,000 × *g*, 4 °C) to pellet any insoluble protein. Samples for SDS-PAGE were taken from the soluble supernatant fraction, or the supernatant was removed and a sample was taken from the insoluble pellet resuspended in 1 mL of water.

## Electronic supplementary material

Below is the link to the electronic supplementary material.

Supplementary Material 1

## Data Availability

All data generated or analysed during this study are included in this published article (and its supplementary information files). Plasmid pSPIH6 and its DNA sequence is available through Addgene (plasmid #190676). DNA sequencing data from pSPIH6-LeuA and pSPIH6-LcnA can be accessed through the DNA Data Bank of Japan (DDBJ) with accession numbers LC747188 and LC747189, respectively (direct web links: http://getentry.ddbj.nig.ac.jp/getentry/na/LC747188/?format=flatfile&filetype=html&trace=true&show_suppressed=false&limit=10 ; http://getentry.ddbj.nig.ac.jp/getentry/na/LC747189/?format=flatfile&filetype=html&trace=true&show_suppressed=false&limit=10 ). Protein sequences of LeuA and LcnA peptides can be accessed through Uniprot (accession numbers P34034 and P0A313, respectively). All DNA and protein sequences are also included in the supplementary information file.

## List of abbreviations

βME: 2-Mercaptoethanol
CBD: Chitin-binding domain
DTT: D,L-dithiothreitol
EDTA: Ethylenediaminetetraacetic acid
GyrA: DNA Gyrase A
His_6_: Polyhistidine tag
HPLC: High performance liquid chromatography
IPTG: Isopropyl β-D-1-thiogalactopyranoside
LB: Luria Broth
LeuA: Leucocin A
LcnA: Lactococcin A
MALDI-TOF MS: Matrix-assisted laser desorption ionization time of flight mass spectrometry
MS: Mass spectrometry
*Mxe*: *Mycobacterium xenopii*
Ni-NTA: Nickel nitrilotriacetic acid
PCR: Polymerase chain reaction
SDS-PAGE: Sodium dodecyl phosphate polyacrylamide gel electrophoresis
SPI: SUMO-peptide-intein
SUMO: Small ubiquitin-like modifier
TCEP: Tris(2-carboxyethyl)phosphine
TFA: Trifluoroacetic acid
