# Supplementary material for: Simplified cloning and isolation of peptides from “sandwiched” SUMO-peptide-intein fusion proteins
Source: BMC Biotechnol. 2023 Apr 5;23:11. doi: 10.1186/s12896-023-00779-5 (PMC10074672; doi:10.1186/s12896-023-00779-5)
Supplement: Supplementary file 1 — Supplementary Material 1 [file 12896_2023_779_MOESM1_ESM.docx]

**Supplementary Information**

Simplified cloning and isolation of peptides from “sandwiched” SUMO-peptide-intein fusion proteins

**Tess Lamer^1^ and John C. Vederas^1*^**

^1^ University of Alberta, Department of Chemistry, T6G 2G2, Edmonton, AB, Canada

*** Corresponding author:**John C. Vederas
[john.vederas@ualberta.ca](mailto:john.vederas@ualberta.ca)

#
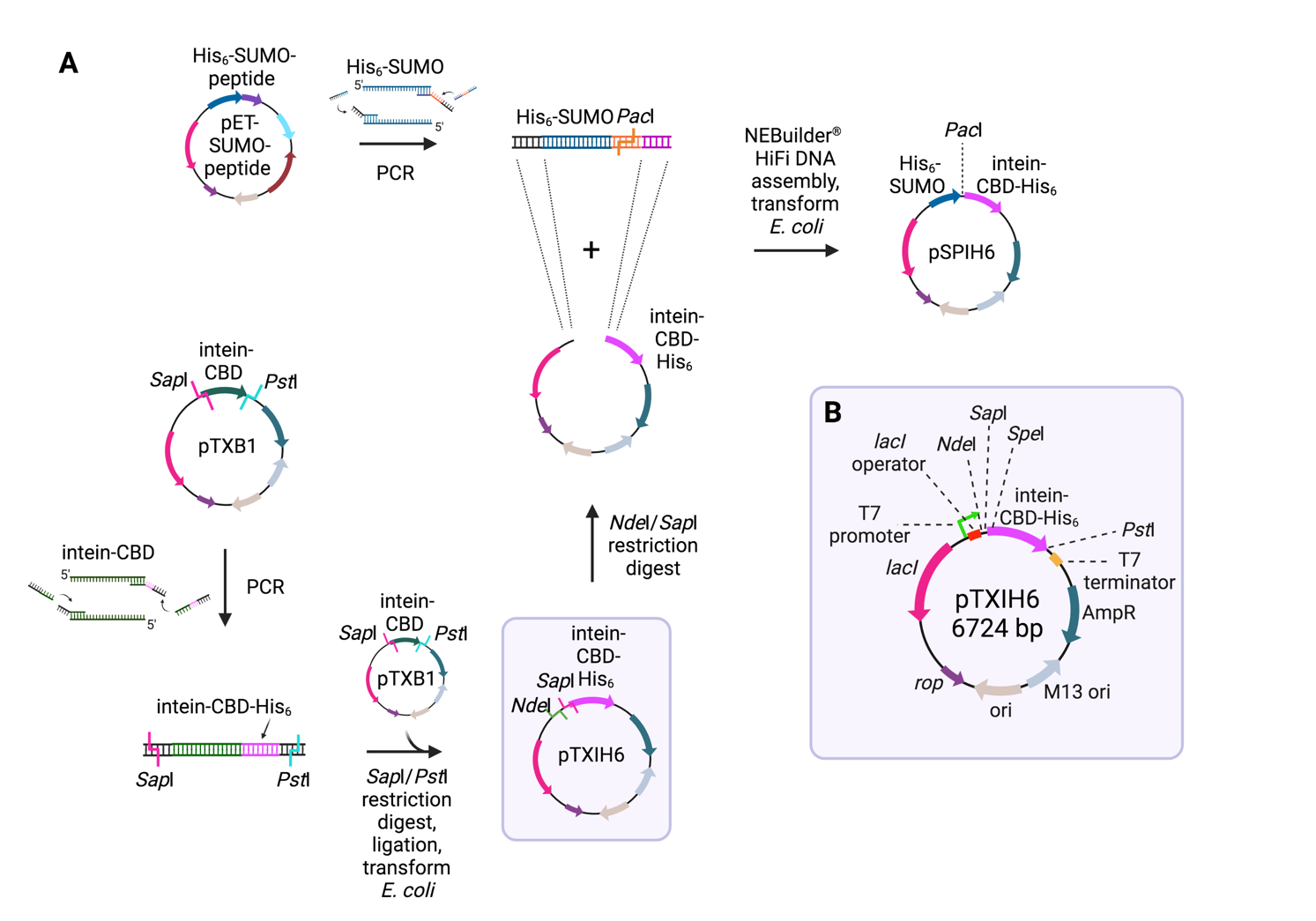
Supplementary Figures

**Supplementary Figure 1.** Outline of cloning procedure to construct pTXIH6 and pSPIH6 plasmids. **(A)** Cloning procedure to construct pSPIH6 from pET-SUMO and pTXB1 vectors. The His_6_-SUMO gene was amplified (top left arrow) using primers that included specific complementary tail sequences for insertion into a *Nde*I and *Sap*I digested pTXIH6 vector. The reverse primer used also included a *Pac*I restriction site directly following the final codon of the SUMO gene. The intein-CBD gene and 5’ *Sap*I and 3’ *Pst*I restriction sites were amplified with PCR (lower left arrow), using a reverse primer to insert a polyhistidine tag at the 3’ end of the gene. The PCR product and pTXB1 were each doubly digested with *Sap*I and *Pst*I to insert the intein-CBD-His_6_ gene into pTXB1 and create plasmid pTXIH6. Then, pTXIH6 was digested with *Nde*I and *Sap*I to create an insertion site for the PCR amplified His_6_-SUMO gene using NEBuilder^®^ HiFi DNA Assembly (top right arrow) to assemble pSPIH6. **(B)** Vector map for pTXIH6, which is identical to pTXB1 (New England BioLabs) except the encoded intein contains an inserted C-terminal polyhistidine tag.


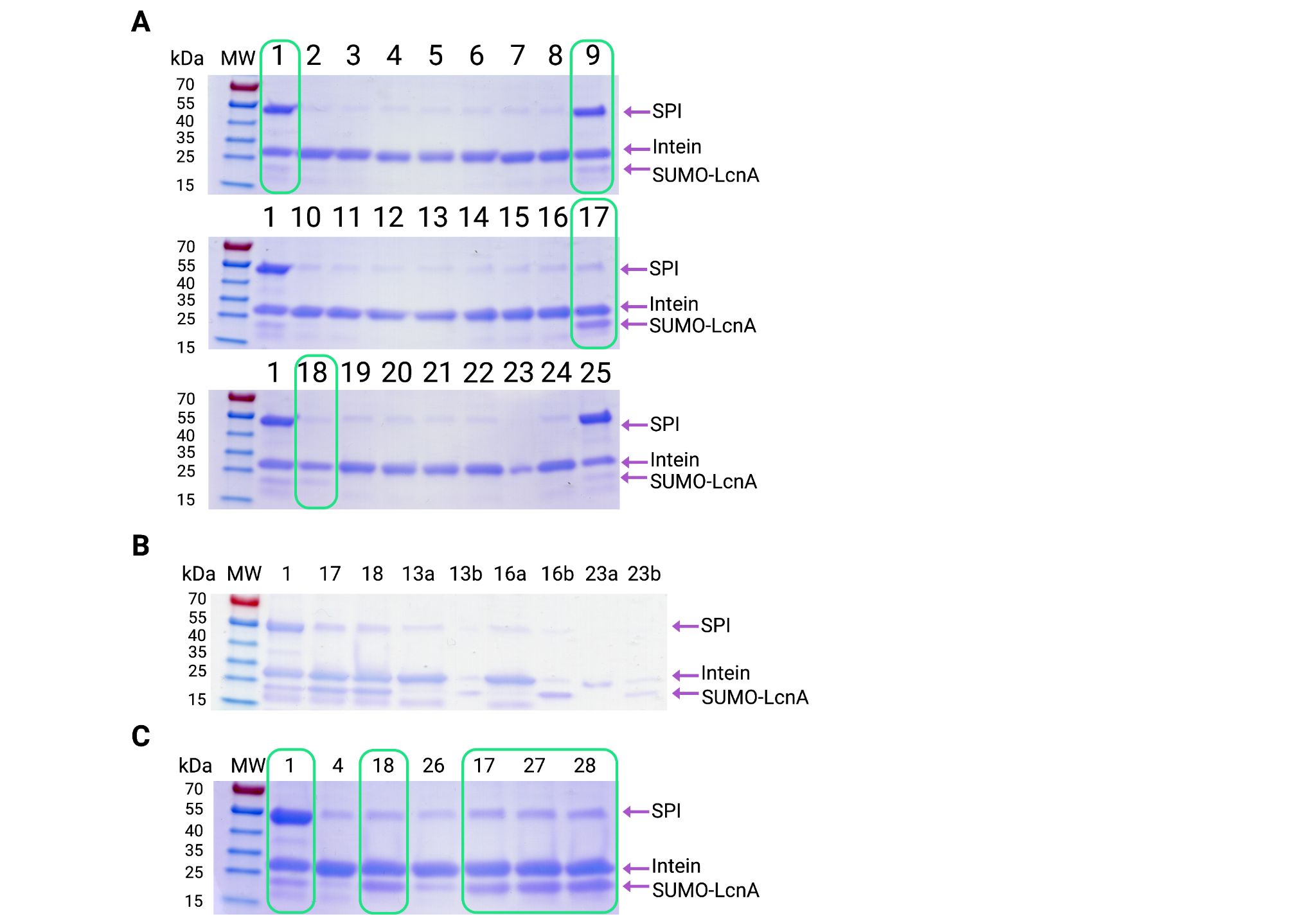


**Supplementary Figure 2.**  Screen of buffer additives to prevent precipitation of SUMO-LcnA during intein cleavage of a SPI fusion protein. **(A)** SDS-PAGE results of a screen of 25 different buffer conditions for intein cleavage. After incubation, samples were centrifuged to pellet any precipitated protein, and SDS-PAGE samples were taken from the soluble supernatant. Each cleavage condition contained a standard base buffer of 50 mM Na_2_HPO_4_, 300 mM NaCl, 150 mM imidazole, pH 8.5. Circled in green are the conditions where no protein precipitation was observed after incubation at 4 °C for 72 h. MW = molecular weight protein ladder; 1 = standard base buffer only; 2 = 100 mM DTT; 3 = 100 mM DTT, 5 mM EDTA; 4 = 100 mM βME, 10 mM TCEP, 5 mM EDTA; 5 = 50 mM DTT, 5 mM EDTA; 6 = 10 mM DTT, 5 mM EDTA; 7 = 50 mM βME, 10 mM TCEP, 5 mM EDTA; 8 = 10 mM βME, 10 mM TCEP, 5 mM EDTA; 9 = 5 mM EDTA; 10 = 100 mM βME, 10 mM TCEP, 5 mM EDTA, 10% (v/v) glycerol; 11 = 100 mM βME, 10 mM TCEP, 5 mM EDTA, 600 mM NaCl total; 12 = 100 mM βME, 10 mM TCEP, 5 mM EDTA, 1 M NaCl total; 13 = 100 mM βME, 10 mM TCEP, 5 mM EDTA, 1.5 M NaCl total; 14 = 100 mM βME, 10 mM TCEP, 5 mM EDTA, 50 mM Na_2_SO_4_; 15 = 100 mM βME, 10 mM TCEP, 5 mM EDTA, 500 mM urea; 16 = 100 mM βME, 10 mM TCEP, 5 mM EDTA, 50 mM (NH_4_)_2_SO_4_; 17 = 100 mM βME, 10 mM TCEP, 5 mM EDTA, 1% (v/v) IGEPAL^®^ CA-630; 18 = 100 mM βME, 10 mM TCEP, 5 mM EDTA, 500 mM arginine hydrochloride; 19 = 100 mM βME, 10 mM TCEP, 200 mM KCl; 20 =100 mM βME, 10 mM TCEP, 100 mM MgSO_4_; 21 = 100 mM βME, 10 mM TCEP, 100 mM MgCl_2_·6H_2_O; 22 = 100 mM βME, 10 mM TCEP, 50 mM Cs_2_SO_4_; 23 = 100 mM βME, 10 mM TCEP, 5 mM EDTA, 500 mM guanidine hydrochloride; 24 = 100 mM βME, 10 mM TCEP, 5 mM EDTA, 125 mM arginine hydrochloride; 25 = 100 mM βME, 10 mM TCEP, 5 mM EDTA, 0.05% SDS (w/v). **(B)** SDS-PAGE results of soluble and insoluble fractions of various cleavage conditions after incubation at 4 °C for 72 h. Conditions 1, 17, and 18 had only soluble fractions (no precipitate observed after incubation and subsequent centrifugation). 13a, 16a, and 23a are soluble supernatants of the respective conditions listed above, after pelleting the protein precipitate by centrifugation. 13b, 16b, and 23b are the insoluble precipitates of respective conditions listed above, resuspended in H_2_O after pelleting by centrifugation. **(C)** SDS-PAGE results of the effects of varying concentrations of arginine hydrochloride and IGEPAL^®^ CA-630 on SUMO-LcnA solubility after intein cleavage. Circled in green are the conditions where no visible protein precipitation was observed after incubation at 4 °C for 72 h. After incubation, samples were centrifuged to pellet any precipitated protein, and SDS-PAGE samples were taken from the soluble supernatant. 1, 4, 17, and 18 correspond to conditions listed above. 26 = 100 mM βME, 10 mM TCEP, 5 mM EDTA, 250 mM arginine hydrochloride; 27 = 100 mM βME, 10 mM TCEP, 5 mM EDTA, 250 mM arginine hydrochloride, 0.5% IGEPAL^®^ CA-630; 28 = 100 mM βME, 10 mM TCEP, 5 mM EDTA, 250 mM arginine hydrochloride, 0.01% IGEPAL^®^ CA-630.

# Supplementary Tables

**Supplementary Table 1.** DNA primer sequences used in this study.

| Primer name | Sequence (5’ to 3’) | Purpose |
| --- | --- | --- |
| Int-F | TATAGCTCTTCCTGCATCACGGGAGATGCACTAGTTG | Forward primer to amplify intein from pTXB1 to construct pTXIH6 |
| Int-R | TACTGCAGTCAGTGATGATGATGATGATGTTGAAGCTGCCACAAGGC | Reverse primer to amplify intein from pTXB1 and add C-terminal polyhistidine tag to construct pTXIH6 |
| Col-Int-F | CTGGACTGGTGCGTTTCTT | Colony PCR forward primer to check for insertion of polyhistidine tag in pTXIH6 |
| Col-Int-R | TCAGTGATGATGATGATGATG | Colony PCR reverse primer to check for insertion of polyhistidine tag in pTXIH6 |
| Int-seq-F-1 | CCGCGAAATTAATACGACTCA | DNA sequencing primer (forward) to check for insertion of polyhistidine tag in pTXIH6 |
| Int-seq-R-1 | GCAAAACCTGCACAGTCGA | DNA sequencing primer (reverse) to check for insertion of polyhistidine tag in pTXIH6 |
| Int-seq-F-2 | ACAAATCCTGGTGTATCCGCTT | DNA sequencing primer (forward) to check for insertion of polyhistidine tag in pTXIH6 |
| Int-seq-R-2 | ATCCGGATATAGTTCCTCCTTT | DNA sequencing primer (forward) to check for insertion of polyhistidine tag in pTXIH6 |
| SUMO-F | TAACTTTAAGAAGGAGATATACATATGGGCAGCAGCCATCATC | Forward primer to amplify His_6_-SUMO from pTXB1-SPI-LeuA and add overlapping end sequence complementary to pTXIH6, to construct pSPIH6 |
| SUMO-R | TAGTGCATCTCCCGTGATGCATTAATTAAACCACCAATCTGTTCTCTGTG | Reverse primer to amplify His_6_-SUMO from pTXB1-SPI-LeuA and add *Pac*I and overlapping end sequence complementary to pTXIH6, to construct pSPIH6 |
| SUMO-Col-F | CCATAAACTGCCAGGAATTGGGG | Colony PCR forward primer to check for insertion of His_6_-SUMO into pTXIH6 to construct pSPIH6 |
| SUMO-Col-R (universal)* | CACGGGATTGCCATGCCGG | Colony PCR reverse primer to check for insertion of His_6_-SUMO into pTXIH6. *Also used as a reverse primer for DNA sequencing of pSPIH6-LeuA and pSPIH6-LcnA, to verify peptide gene insertion and sequence* |
| LeuA-G-F** | ACAGAGAACAGATTGGTGGTAAATATTATGGCAACGGTGT | Forward primer to amplify LeuA from pTXB1-SPI-LeuA and add overlapping end sequence complementary to pSPIH6 for insertion into *Pac*I site in pSPIH6. **The underlined 20 bp create an overlapping sequence with the 3’ end of the SUMO gene in the PCR product, which allows for seamless insertion of the amplified LeuA gene into a *Pac*I digested pSPIH6 with NEBuilder^®^ HiFi DNA Assembly. This underlined sequence is universal and can be appended to any DNA primer sequence for insertion into pSPIH6** |
| LeuA-G-R** | AGTGCATCTCCCGTGATGCACCAAAAGC | Reverse primer to amplify LeuA from pTXB1-SPI-LeuA and add overlapping end sequence complementary to pSPIH6 for insertion into *Pac*I site in pSPIH6. **The underlined 20 bp create an overlapping sequence with the 5’ end of the intein gene in the PCR product, which allows for seamless insertion of the amplified LeuA gene into a *Pac*I digested pSPIH6 with NEBuilder^®^ HiFi DNA Assembly. This underlined sequence is universal and can be appended to any DNA primer sequence for insertion into pSPIH6** |
| LcnA-G-F** | ACAGAGAACAGATTGGTGGTAAACTGACCTTTATTCAGAG | Forward primer to amplify LcnA from pTXB1-SPI-LcnA and add overlapping end sequence complementary to pSPIH6 for insertion into *Pac*I site in pSPIH6. **The underlined 20 bp create an overlapping sequence with the 3’ end of the SUMO gene in the PCR product, which allows for seamless insertion of the amplified LeuA gene into a *Pac*I digested pSPIH6 with NEBuilder^®^ HiFi DNA Assembly. This underlined sequence is universal and can be appended to any DNA primer sequence for insertion into pSPIH6** |
| LcnA-G-R** | AGTGCATCTCCCGTGATGCAATGATGCA | Reverse primer to amplify LcnA from pTXB1-SPI-LcnA and add overlapping end sequence complementary to pSPIH6 for insertion into *Pac*I site in pSPIH6. **The underlined 20 bp create an overlapping sequence with the 5’ end of the intein gene in the PCR product, which allows for seamless insertion of the amplified LeuA gene into a *Pac*I digested pSPIH6 with NEBuilder^®^ HiFi DNA Assembly. This underlined sequence is universal and can be appended to any DNA primer sequence for insertion into pSPIH6** |
| TL-pTXB1-F (universal)* | TAATACGACTCACTATAGGGG | *DNA Sequencing primer (forward) to confirm peptide gene insertion and sequence in pSPIH6-LeuA and pSPIH6-LcnA* |
| SPI-col-F (universal)* | GACCCCTGAAGATTTGGACATGGAG | *Forward colony PCR primer to check for peptide gene insertion in pSPIH6. Without insert, the size of the colony PCR product is 123 bp* |
| SPI-col-R (universal)* | TACCGACTCGCCCTCGGG | *Reverse colony PCR primer to check for peptide insertion in pSPIH6. Without insert, the size of the colony PCR product is 123 bp* |

*These universal primers can be used for colony PCR and DNA sequencing of any inserted peptide sequence into pSPIH6.

**Underlined universal sequences in these primers create 20 bp overlap sequences with either the 3’ end of the SUMO gene (forward primers) or the 5’ end of the intein gene (reverse primers) to allow for insertion of the PCR amplified peptide gene into a *Pac*I digested pSPIH6 with NEBuilder^®^ HiFi DNA Assembly.

**Supplementary Table 2.** Protein sequences and monoisotopic masses.

| **Protein** | **Amino acid sequence** | **Predicted monoisotopic mass** |
| --- | --- | --- |
| **His_6_-SUMO** | fMGSSHHHHHHGSGLVPRGSASMSDSEVNQEAKPEVKPEVKPETHINLKVSDGSSEIFFKIKKTTPLRRLMEAFAKRQGKEMDSLRFLYDGIRIQADQTPEDLDMEDNDIIEAHREQIGG | 13435.6  (13276.6)* |
| **Intein-His_6_** | CITGDALVALPEGESVRIADIVPGARPNSDNAIDLKVLDRHGNPVLADRLFHSGEHPVYTVRTVEGLRVTGTANHPLLCLVDVAGVPTLLWKLIDEIKPGDYAVIQRSAFSVDCAGFARGKPEFAPTTYTVGVPGLVRFLEAHHRDPDAQAIADELTDGRFYYAKVASVTDAGVQPVYSLRVDTADHAFITNGFVSHATGLTGLNSGLTTNPGVSAWQVNTAYTAGQLVTYNGKTYKCLQPHTSLAGWEPSNVPALWQLQHHHHHH | 28663.6 |
| **LcnA** | KLTFIQSTAAGDLYYNTNTHKYVYQQTQNAFGAAANTIVNGWMGGAAGGFGLHH | 5774.8 |
| **LeuA** | KYYGNGVHCTKSGCSVNWGEAFSAGVHRLANGGNGFW | 3929.8 |
| **His_6_-SUMO-**  **LcnA** | fMGSSHHHHHHGSGLVPRGSASMSDSEVNQEAKPEVKPEVKPETHINLKVSDGSSEIFFKIKKTTPLRRLMEAFAKRQGKEMDSLRFLYDGIRIQADQTPEDLDMEDNDIIEAHREQIGGKLTFIQSTAAGDLYYNTNTHKYVYQQTQNAFGAAANTIVNGWMGGAAGGFGLHH | 19192.4  (19033.4)* |
| **His_6_-SUMO- LeuA** | fMGSSHHHHHHGSGLVPRGSASMSDSEVNQEAKPEVKPEVKPETHINLKVSDGSSEIFFKIKKTTPLRRLMEAFAKRQGKEMDSLRFLYDGIRIQADQTPEDLDMEDNDIIEAHREQIGGKYYGNGVHCT KSGCSVNWGEAFSAGVHRLANGGNGFW | 17347.4  (17188.4)* |
| **His_6_-SUMO-**  **LcnA- Intein-His_6_** | fMGSSHHHHHHGSGLVPRGSASMSDSEVNQEAKPEVKPEVKPETHINLKVSDGSSEIFFKIKKTTPLRRLMEAFAKRQGKEMDSLRFLYDGIRIQADQTPEDLDMEDNDIIEAHREQIGGKLTFIQSTAAGDLYYNTNTHKYVYQQTQNAFGAAANTIVNGWMGGAAGGFGLHHCITGDALVALPEGESVRIADIVPGARPNSDNAIDLKVLDRHGNPVLADRLFHSGEHPVYTVRTVEGLRVTGTANHPLLCLVDVAGVPTLLWKLIDEIKPGDYAVIQRSAFSVDCAGFARGKPEFAPTTYTVGVPGLVRFLEAHHRDPDAQAIADELTDGRFYYAKVASVTDAGVQPVYSLRVDTADHAFITNGFVSHATGLTGLNSGLTTNPGVSAWQVNTAYTAGQLVTYNGKTYKCLQPHTSLAGWEPSNVPAL WQLQHHHHHH | 47837.9  (47678.9)* |
| **His_6_-SUMO- LeuA- Intein-His_6_** | fMGSSHHHHHHGSGLVPRGSASMSDSEVNQEAKPEVKPEVKPETHINLKVSDGSSEIFFKIKKTTPLRRLMEAFAKRQGKEMDSLRFLYDGIRIQADQTPEDLDMEDNDIIEAHREQIGGKYYGNGVHCTKSGCSVNWGEAFSAGVHRLANGGNGFWCITGDALVALPEGESVRIADIVPGARPNSDNAIDLKVLDRHGNPVLADRLFHSGEHPVYTVRTVEGLRVTGTANHPLLCLVDVAGVPTLLWKLIDEIKPGDYAVIQRSAFSVDCAGFARGKPEFAPTTYTVGVPGLVRFLEAHHRDPDAQAIADELTDGRFYYAKVASVTDAGVQPVYSLRVDTADHAFITNGFVSHATGLTGLNSGLTTNPGVSAWQVNTAYTAGQLVTYNGKTYKCLQPHTSLAGWEPSNVPALWQLQHHHHHH | 45993.0  (45833.9)* |

***Predicted monoisotopic mass without N-terminal formyl methionine.**

# Supplementary Data

**DNA Sequences:**

**Intein sequence in pTXB1 (blue = *Mxe* GyrA mutated intein with 3’ Ala substitution, black = linker sequence, red = CBD, bold = stop codon):**

ATGGCTAGCTCGCGAGTCGACGGCGGCCGCGAATTCCTCGAGGGCTCTTCCTGCATCACGGGAGATGCACTAGTTGCCCTACCCGAGGGCGAGTCGGTACGCATCGCCGACATCGTGCCGGGTGCGCGGCCCAACAGTGACAACGCCATCGACCTGAAAGTCCTTGACCGGCATGGCAATCCCGTGCTCGCCGACCGGCTGTTCCACTCCGGCGAGCATCCGGTGTACACGGTGCGTACGGTCGAAGGTCTGCGTGTGACGGGCACCGCGAACCACCCGTTGTTGTGTTTGGTCGACGTCGCCGGGGTGCCGACCCTGCTGTGGAAGCTGATCGACGAAATCAAGCCGGGCGATTACGCGGTGATTCAACGCAGCGCATTCAGCGTCGACTGTGCAGGTTTTGCCCGCGGGAAACCCGAATTTGCGCCCACAACCTACACAGTCGGCGTCCCTGGACTGGTGCGTTTCTTGGAAGCACACCACCGAGACCCGGACGCCCAAGCTATCGCCGACGAGCTGACCGACGGGCGGTTCTACTACGCGAAAGTCGCCAGTGTCACCGACGCCGGCGTGCAGCCGGTGTATAGCCTTCGTGTCGACACGGCAGACCACGCGTTTATCACGAACGGGTTCGTCAGCCACGCTACTGGCCTCACCGGTCTGAACTCAGGCCTCACGACAAATCCTGGTGTATCCGCTTGGCAGGTCAACACAGCTTATACTGCGGGACAATTGGTCACATATAACGGCAAGACGTATAAATGTTTGCAGCCCCACACCTCCTTGGCAGGATGGGAACCATCCAACGTTCCTGCCTTGTGGCAGCTTCAA**TGA**

**pTXB1 (*Sap*I and *Pst*I highlighted, intein sequence in bold, *intein catalytic Cys*):**

AACTACGTCAGGTGGCACTTTTCGGGGAAATGTGCGCGGAACCCCTATTTGTTTATTTTTCTAAATACATTCAAATATGTATCCGCTCATGAGACAATAACCCTGATAAATGCTTCAATAATATTGAAAAAGGAAGAGTATGAGTATTCAACATTTCCGTGTCGCCCTTATTCCCTTTTTTGCGGCATTTTGCCTTCCTGTTTTTGCTCACCCAGAAACGCTGGTGAAAGTAAAAGATGCTGAAGATCAGTTGGGTGCACGAGTGGGTTACATCGAACTGGATCTCAACAGCGGTAAGATCCTTGAGAGTTTTCGCCCCGAAGAACGTTCTCCAATGATGAGCACTTTTAAAGTTCTGCTATGTGGCGCGGTATTATCCCGTGTTGACGCCGGGCAAGAGCAACTCGGTCGCCGCATACACTATTCTCAGAATGACTTGGTTGAGTACTCACCAGTCACAGAAAAGCATCTTACGGATGGCATGACAGTAAGAGAATTATGCAGTGCTGCCATAACCATGAGTGATAACACTGCGGCCAACTTACTTCTGACAACGATCGGAGGACCGAAGGAGCTAACCGCTTTTTTGCACAACATGGGGGATCATGTAACTCGCCTTGATCGTTGGGAACCGGAGCTGAATGAAGCCATACCAAACGACGAGCGTGACACCACGATGCCTGTAGCAATGGCAACAACGTTGCGCAAACTATTAACTGGCGAACTACTTACTCTAGCTTCCCGGCAACAATTAATAGACTGGATGGAGGCGGATAAAGTTGCAGGACCACTTCTGCGCTCGGCCCTTCCGGCTGGCTGGTTTATTGCTGATAAATCTGGAGCCGGTGAGCGTGGGTCTCGCGGTATCATTGCAGCACTGGGGCCAGATGGTAAGCCCTCCCGTATCGTAGTTATCTACACGACGGGGAGTCAGGCAACTATGGATGAACGAAATAGACAGATCGCTGAGATAGGTGCCTCACTGATTAAGCATTGGTAACTGTCAGACCAAGTTTACTCATATATACTTTAGATTGATTTACCCCGGTTGATAATCAGAAAAGCCCCAAAAACAGGAAGATTGTATAAGCAAATATTTAAATTGTAAACGTTAATATTTTGTTAAAATTCGCGTTAAATTTTTGTTAAATCAGCTCATTTTTTAACCAATAGGCCGAAATCGGCAAAATCCCTTATAAATCAAAAGAATAGCCCGAGATAGGGTTGAGTGTTGTTCCAGTTTGGAACAAGAGTCCACTATTAAAGAACGTGGACTCCAACGTCAAAGGGCGAAAAACCGTCTATCAGGGCGATGGCCCACTACGTGAACCATCACCCAAATCAAGTTTTTTGGGGTCGAGGTGCCGTAAAGCACTAAATCGGAACCCTAAAGGGAGCCCCCGATTTAGAGCTTGACGGGGAAAGCCGGCGAACGTGGCGAGAAAGGAAGGGAAGAAAGCGAAAGGAGCGGGCGCTAGGGCGCTGGCAAGTGTAGCGGTCACGCTGCGCGTAACCACCACACCCGCCGCGCTTAATGCGCCGCTACAGGGCGCGTAAAAGGATCTAGGTGAAGATCCTTTTTGATAATCTCATGACCAAAATCCCTTAACGTGAGTTTTCGTTCCACTGAGCGTCAGACCCCGTAGAAAAGATCAAAGGATCTTCTTGAGATCCTTTTTTTCTGCGCGTAATCTGCTGCTTGCAAACAAAAAAACCACCGCTACCAGCGGTGGTTTGTTTGCCGGATCAAGAGCTACCAACTCTTTTTCCGAAGGTAACTGGCTTCAGCAGAGCGCAGATACCAAATACTGTCCTTCTAGTGTAGCCGTAGTTAGGCCACCACTTCAAGAACTCTGTAGCACCGCCTACATACCTCGCTCTGCTAATCCTGTTACCAGTGGCTGCTGCCAGTGGCGATAAGTCGTGTCTTACCGGGTTGGACTCAAGACGATAGTTACCGGATAAGGCGCAGCGGTCGGGCTGAACGGGGGGTTCGTGCACACAGCCCAGCTTGGAGCGAACGACCTACACCGAACTGAGATACCTACAGCGTGAGCTATGAGAAAGCGCCACGCTTCCCGAAGGGAGAAAGGCGGACAGGTATCCGGTAAGCGGCAGGGTCGGAACAGGAGAGCGCACGAGGGAGCTTCCAGGGGGAAACGCCTGGTATCTTTATAGTCCTGTCGGGTTTCGCCACCTCTGACTTGAGCGTCGATTTTTGTGATGCTCGTCAGGGGGGCGGAGCCTATGGAAAAACGCCAGCAACGCGGCCTTTTTACGGTTCCTGGCCTTTTGCTGGCCTTTTGCTCACATGTTCTTTCCTGCGTTATCCCCTGATTCTGTGGATAACCGTATTACCGCCTTTGAGTGAGCTGATACCGCTCGCCGCAGCCGAACGACCGAGCGCAGCGAGTCAGTGAGCGAGGAAGCTATGGTGCACTCTCAGTACAATCTGCTCTGATGCCGCATAGTTAAGCCAGTATACACTCCGCTATCGCTACGTGACTGGGTCATGGCTGCGCCCCGACACCCGCCAACACCCGCTGACGCGCCCTGACGGGCTTGTCTGCTCCCGGCATCCGCTTACAGACAAGCTGTGACCGTCTCCGGGAGCTGCATGTGTCAGAGGTTTTCACCGTCATCACCGAAACGCGCGAGGCAGCTGCGGTAAAGCTCATCAGCGTGGTCGTGCAGCGATTCACAGATGTCTGCCTGTTCATCCGCGTCCAGCTCGTTGAGTTTCTCCAGAAGCGTTAATGTCTGGCTTCTGATAAAGCGGGCCATGTTAAGGGCGGTTTTTTCCTGTTTGGTCACTGATGCCTCCGTGTAAGGGGGATTTCTGTTCATGGGGGTAATGATACCGATGAAACGAGAGAGGATGCTCACGATACGGGTTACTGATGATGAACATGCCCGGTTACTGGAACGTTGTGAGGGTAAACAACTGGCGGTATGGATGCGGCGGGACCAGAGAAAAATCACTCAGGGTCAATGCCAGCCGAACGCCAGCAAGACGTAGCCCAGCGCGTCGGCCGCCATGCCGGCGATAATGGCCTGCTTCTCGCCGAAACGTTTGGTGGCGGGACCAGTGACGAAGGCTTGAGCGAGGGCGTGCAAGATTCCGAATACCGCAAGCGACAGGCCGATCATCGTCGCGCTCCAGCGAAAGCGGTCCTCGCCGAAAATGACCCAGAGCGCTGCCGGCACCTGTCCTACGAGTTGCATGATAAAGAAGACAGTCATAAGTGCGGCGACGATAGTCATGCCCCGCGCCCACCGGAAGGAGCTGACTGGGTTGAAGGCTCTCAAGGGCATCGGTCGAGATCCCGGTGCCTAATGAGTGAGCTAACTTACATTAATTGCGTTGCGCTCACTGCCCGCTTTCCAGTCGGGAAACCTGTCGTGCCAGCTGCATTAATGAATCGGCCAACGCGCGGGGAGAGGCGGTTTGCGTATTGGGCGCCAGGGTGGTTTTTCTTTTCACCAGTGAGACGGGCAACAGCTGATTGCCCTTCACCGCCTGGCCCTGAGAGAGTTGCAGCAAGCGGTCCACGCTGGTTTGCCCCAGCAGGCGAAAATCCTGTTTGATGGTGGTTAACGGCGGGATATAACATGAGCTGTCTTCGGTATCGTCGTATCCCACTACCGAGATATCCGCACCAACGCGCAGCCCGGACTCGGTAATGGCGCGCATTGCGCCCAGCGCCATCTGATCGTTGGCAACCAGCATCGCAGTGGGAACGATGCCCTCATTCAGCATTTGCATGGTTTGTTGAAAACCGGACATGGCACTCCAGTCGCCTTCCCGTTCCGCTATCGGCTGAATTTGATTGCGAGTGAGATATTTATGCCAGCCAGCCAGACGCAGACGCGCCGAGACAGAACTTAATGGGCCCGCTAACAGCGCGATTTGCTGGTGACCCAATGCGACCAGATGCTCCACGCCCAGTCGCGTACCGTCTTCATGGGAGAAAATAATACTGTTGATGGGTGTCTGGTCAGAGACATCAAGAAATAACGCCGGAACATTAGTGCAGGCAGCTTCCACAGCAATGGCATCCTGGTCATCCAGCGGATAGTTAATGATCAGCCCACTGACGCGTTGCGCGAGAAGATTGTGCACCGCCGCTTTACAGGCTTCGACGCCGCTTCGTTCTACCATCGACACCACCACGCTGGCACCCAGTTGATCGGCGCGAGATTTAATCGCCGCGACAATTTGCGACGGCGCGTGCAGGGCCAGACTGGAGGTGGCAACGCCAATCAGCAACGACTGTTTGCCCGCCAGTTGTTGTGCCACGCGGTTGGGAATGTAATTCAGCTCCGCCATCGCCGCTTCCACTTTTTCCCGCGTTTTCGCAGAAACGTGGCTGGCCTGGTTCACCACGCGGGAAACGGTCTGATAAGAGACACCGGCATACTCTGCGACATCGTATAACGTTACTGGTTTCACATTCACCACCCTGAATTGACTCTCTTCCGGGCGCTATCATGCCATACCGCGAAAGGTTTTGCGCCATTCGATGGTGTCCGGGATCTCGACGCTCTCCCTTATGCGACTCCTGCATTAGGAAGCAGCCCAGTAGTAGGTTGAGGCCGTTGAGCACCGCCGCCGCAAGGAATGGTGCATGCCGGCATGCCGCCCTTTCGTCTTCAAGAATTAATTCCCAATTCCCCAGGCATCAAATAAAACGAAAGGCTCAGTCGAAAGACTGGGCCTTTCGTTTTATCTGTTGTTTGTCGGTGAACGCTCTCCTGAGTAGGACAAATCCGCCGGGAGCGGATTTGAACGTTGCGAAGCAACGGCCCGGAGGGTGGCGGGCAGGACGCCCGCCATAAACTGCCAGGAATTAATTCCCCAGGCATCAAATAAAACGAAAGGCTCAGTCGAAAGACTGGGCCTTTCGTTTTATCTGTTGTTTGTCGGTGAACGCTCTCCTGAGTAGGACAAATCCGCCGGGAGCGGATTTGAACGTTGCGAAGCAACGGCCCGGAGGGTGGCGGGCAGGACGCCCGCCATAAACTGCCAGGAATTAATTCCCCAGGCATCAAATAAAACGAAAGGCTCAGTCGAAAGACTGGGCCTTTCGTTTTATCTGTTGTTTGTCGGTGAACGCTCTCCTGAGTAGGACAAATCCGCCGGGAGCGGATTTGAACGTTGCGAAGCAACGGCCCGGAGGGTGGCGGGCAGGACGCCCGCCATAAACTGCCAGGAATTAATTCCCCAGGCATCAAATAAAACGAAAGGCTCAGTCGAAAGACTGGGCCTTTCGTTTTATCTGTTGTTTGTCGGTGAACGCTCTCCTGAGTAGGACAAATCCGCCGGGAGCGGATTTGAACGTTGCGAAGCAACGGCCCGGAGGGTGGCGGGCAGGACGCCCGCCATAAACTGCCAGGAATTAATTCCCCAGGCATCAAATAAAACGAAAGGCTCAGTCGAAAGACTGGGCCTTTCGTTTTATCTGTTGTTTGTCGGTGAACGCTCTCCTGAGTAGGACAAATCCGCCGGGAGCGGATTTGAACGTTGCGAAGCAACGGCCCGGAGGGTGGCGGGCAGGACGCCCGCCATAAACTGCCAGGAATTGGGGATCGGAATTAATTCCCGGTTTAAACCGGGGATCTCGATCCCGCGAAATTAATACGACTCACTATAGGGGAATTGTGAGCGGATAACAATTCCCCTCTAGAAATAATTTTGTTTAACTTTAAGAAGGAGATATACAT**ATGGCTAGCTCGCGAGTCGACGGCGGCCGCGAATTCCTCGAGGGCTCTTCC*TGC*ATCACGGGAGATGCACTAGTTGCCCTACCCGAGGGCGAGTCGGTACGCATCGCCGACATCGTGCCGGGTGCGCGGCCCAACAGTGACAACGCCATCGACCTGAAAGTCCTTGACCGGCATGGCAATCCCGTGCTCGCCGACCGGCTGTTCCACTCCGGCGAGCATCCGGTGTACACGGTGCGTACGGTCGAAGGTCTGCGTGTGACGGGCACCGCGAACCACCCGTTGTTGTGTTTGGTCGACGTCGCCGGGGTGCCGACCCTGCTGTGGAAGCTGATCGACGAAATCAAGCCGGGCGATTACGCGGTGATTCAACGCAGCGCATTCAGCGTCGACTGTGCAGGTTTTGCCCGCGGGAAACCCGAATTTGCGCCCACAACCTACACAGTCGGCGTCCCTGGACTGGTGCGTTTCTTGGAAGCACACCACCGAGACCCGGACGCCCAAGCTATCGCCGACGAGCTGACCGACGGGCGGTTCTACTACGCGAAAGTCGCCAGTGTCACCGACGCCGGCGTGCAGCCGGTGTATAGCCTTCGTGTCGACACGGCAGACCACGCGTTTATCACGAACGGGTTCGTCAGCCACGCTACTGGCCTCACCGGTCTGAACTCAGGCCTCACGACAAATCCTGGTGTATCCGCTTGGCAGGTCAACACAGCTTATACTGCGGGACAATTGGTCACATATAACGGCAAGACGTATAAATGTTTGCAGCCCCACACCTCCTTGGCAGGATGGGAACCATCCAACGTTCCTGCCTTGTGGCAGCTTCAATGA**CTGCAGGAAGGGGATCCGGCTGCTAACAAAGCCCGAAAGGAAGCTGAGTTGGCTGCTGCCACCGCTGAGCAATAACTAGCATAACCCCTTGGGGCCTCTAAACGGGTCTTGAGGGGTTTTTTGCTGAAAGGAGGAACTATATCCGGAT

**pTXIH6 (*Nde*I, *Sap*I, and *Pst*I highlighted, intein sequence in bold, His_6_ underlined, *intein catalytic Cys*):**

AACTACGTCAGGTGGCACTTTTCGGGGAAATGTGCGCGGAACCCCTATTTGTTTATTTTTCTAAATACATTCAAATATGTATCCGCTCATGAGACAATAACCCTGATAAATGCTTCAATAATATTGAAAAAGGAAGAGTATGAGTATTCAACATTTCCGTGTCGCCCTTATTCCCTTTTTTGCGGCATTTTGCCTTCCTGTTTTTGCTCACCCAGAAACGCTGGTGAAAGTAAAAGATGCTGAAGATCAGTTGGGTGCACGAGTGGGTTACATCGAACTGGATCTCAACAGCGGTAAGATCCTTGAGAGTTTTCGCCCCGAAGAACGTTTCCCAATGATGAGCACTTTTAAAGTTCTGCTATGTGGCGCGGTATTATCCCGTGTTGACGCCGGGCAAGAGCAACTCGGTCGCCGCATACACTATTCTCAGAATGACTTGGTTGAGTACTCACCAGTCACAGAAAAGCATCTTACGGATGGCATGACAGTAAGAGAATTATGCAGTGCTGCCATAACCATGAGTGATAACACTGCGGCCAACTTACTTCTGACAACGATCGGAGGACCGAAGGAGCTAACCGCTTTTTTGCACAACATGGGGGATCATGTAACTCGCCTTGATCGTTGGGAACCGGAGCTGAATGAAGCCATACCAAACGACGAGCGTGACACCACGATGCCTGTAGCAATGGCAACAACGTTGCGCAAACTATTAACTGGCGAACTACTTACTCTAGCTTCCCGGCAACAATTAATAGACTGGATGGAGGCGGATAAAGTTGCAGGACCACTTCTGCGCTCGGCCCTTCCGGCTGGCTGGTTTATTGCTGATAAATCTGGAGCCGGTGAGCGTGGGTCTCGCGGTATCATTGCAGCACTGGGGCCAGATGGTAAGCCCTCCCGTATCGTAGTTATCTACACGACGGGGAGTCAGGCAACTATGGATGAACGAAATAGACAGATCGCTGAGATAGGTGCCTCACTGATTAAGCATTGGTAACTGTCAGACCAAGTTTACTCATATATACTTTAGATTGATTTACCCCGGTTGATAATCAGAAAAGCCCCAAAAACAGGAAGATTGTATAAGCAAATATTTAAATTGTAAACGTTAATATTTTGTTAAAATTCGCGTTAAATTTTTGTTAAATCAGCTCATTTTTTAACCAATAGGCCGAAATCGGCAAAATCCCTTATAAATCAAAAGAATAGCCCGAGATAGGGTTGAGTGTTGTTCCAGTTTGGAACAAGAGTCCACTATTAAAGAACGTGGACTCCAACGTCAAAGGGCGAAAAACCGTCTATCAGGGCGATGGCCCACTACGTGAACCATCACCCAAATCAAGTTTTTTGGGGTCGAGGTGCCGTAAAGCACTAAATCGGAACCCTAAAGGGAGCCCCCGATTTAGAGCTTGACGGGGAAAGCCGGCGAACGTGGCGAGAAAGGAAGGGAAGAAAGCGAAAGGAGCGGGCGCTAGGGCGCTGGCAAGTGTAGCGGTCACGCTGCGCGTAACCACCACACCCGCCGCGCTTAATGCGCCGCTACAGGGCGCGTAAAAGGATCTAGGTGAAGATCCTTTTTGATAATCTCATGACCAAAATCCCTTAACGTGAGTTTTCGTTCCACTGAGCGTCAGACCCCGTAGAAAAGATCAAAGGATCTTCTTGAGATCCTTTTTTTCTGCGCGTAATCTGCTGCTTGCAAACAAAAAAACCACCGCTACCAGCGGTGGTTTGTTTGCCGGATCAAGAGCTACCAACTCTTTTTCCGAAGGTAACTGGCTTCAGCAGAGCGCAGATACCAAATACTGTCCTTCTAGTGTAGCCGTAGTTAGGCCACCACTTCAAGAACTCTGTAGCACCGCCTACATACCTCGCTCTGCTAATCCTGTTACCAGTGGCTGCTGCCAGTGGCGATAAGTCGTGTCTTACCGGGTTGGACTCAAGACGATAGTTACCGGATAAGGCGCAGCGGTCGGGCTGAACGGGGGGTTCGTGCACACAGCCCAGCTTGGAGCGAACGACCTACACCGAACTGAGATACCTACAGCGTGAGCTATGAGAAAGCGCCACGCTTCCCGAAGGGAGAAAGGCGGACAGGTATCCGGTAAGCGGCAGGGTCGGAACAGGAGAGCGCACGAGGGAGCTTCCAGGGGGAAACGCCTGGTATCTTTATAGTCCTGTCGGGTTTCGCCACCTCTGACTTGAGCGTCGATTTTTGTGATGCTCGTCAGGGGGGCGGAGCCTATGGAAAAACGCCAGCAACGCGGCCTTTTTACGGTTCCTGGCCTTTTGCTGGCCTTTTGCTCACATGTTCTTTCCTGCGTTATCCCCTGATTCTGTGGATAACCGTATTACCGCCTTTGAGTGAGCTGATACCGCTCGCCGCAGCCGAACGACCGAGCGCAGCGAGTCAGTGAGCGAGGAAGCTATGGTGCACTCTCAGTACAATCTGCTCTGATGCCGCATAGTTAAGCCAGTATACACTCCGCTATCGCTACGTGACTGGGTCATGGCTGCGCCCCGACACCCGCCAACACCCGCTGACGCGCCCTGACGGGCTTGTCTGCTCCCGGCATCCGCTTACAGACAAGCTGTGACCGTCTCCGGGAGCTGCATGTGTCAGAGGTTTTCACCGTCATCACCGAAACGCGCGAGGCAGCTGCGGTAAAGCTCATCAGCGTGGTCGTGCAGCGATTCACAGATGTCTGCCTGTTCATCCGCGTCCAGCTCGTTGAGTTTCTCCAGAAGCGTTAATGTCTGGCTTCTGATAAAGCGGGCCATGTTAAGGGCGGTTTTTTCCTGTTTGGTCACTGATGCCTCCGTGTAAGGGGGATTTCTGTTCATGGGGGTAATGATACCGATGAAACGAGAGAGGATGCTCACGATACGGGTTACTGATGATGAACATGCCCGGTTACTGGAACGTTGTGAGGGTAAACAACTGGCGGTATGGATGCGGCGGGACCAGAGAAAAATCACTCAGGGTCAATGCCAGCCGAACGCCAGCAAGACGTAGCCCAGCGCGTCGGCCGCCATGCCGGCGATAATGGCCTGCTTCTCGCCGAAACGTTTGGTGGCGGGACCAGTGACGAAGGCTTGAGCGAGGGCGTGCAAGATTCCGAATACCGCAAGCGACAGGCCGATCATCGTCGCGCTCCAGCGAAAGCGGTCCTCGCCGAAAATGACCCAGAGCGCTGCCGGCACCTGTCCTACGAGTTGCATGATAAAGAAGACAGTCATAAGTGCGGCGACGATAGTCATGCCCCGCGCCCACCGGAAGGAGCTGACTGGGTTGAAGGCTCTCAAGGGCATCGGTCGAGATCCCGGTGCCTAATGAGTGAGCTAACTTACATTAATTGCGTTGCGCTCACTGCCCGCTTTCCAGTCGGGAAACCTGTCGTGCCAGCTGCATTAATGAATCGGCCAACGCGCGGGGAGAGGCGGTTTGCGTATTGGGCGCCAGGGTGGTTTTTCTTTTCACCAGTGAGACGGGCAACAGCTGATTGCCCTTCACCGCCTGGCCCTGAGAGAGTTGCAGCAAGCGGTCCACGCTGGTTTGCCCCAGCAGGCGAAAATCCTGTTTGATGGTGGTTAACGGCGGGATATAACATGAGCTGTCTTCGGTATCGTCGTATCCCACTACCGAGATATCCGCACCAACGCGCAGCCCGGACTCGGTAATGGCGCGCATTGCGCCCAGCGCCATCTGATCGTTGGCAACCAGCATCGCAGTGGGAACGATGCCCTCATTCAGCATTTGCATGGTTTGTTGAAAACCGGACATGGCACTCCAGTCGCCTTCCCGTTCCGCTATCGGCTGAATTTGATTGCGAGTGAGATATTTATGCCAGCCAGCCAGACGCAGACGCGCCGAGACAGAACTTAATGGGCCCGCTAACAGCGCGATTTGCTGGTGACCCAATGCGACCAGATGCTCCACGCCCAGTCGCGTACCGTCTTCATGGGAGAAAATAATACTGTTGATGGGTGTCTGGTCAGAGACATCAAGAAATAACGCCGGAACATTAGTGCAGGCAGCTTCCACAGCAATGGCATCCTGGTCATCCAGCGGATAGTTAATGATCAGCCCACTGACGCGTTGCGCGAGAAGATTGTGCACCGCCGCTTTACAGGCTTCGACGCCGCTTCGTTCTACCATCGACACCACCACGCTGGCACCCAGTTGATCGGCGCGAGATTTAATCGCCGCGACAATTTGCGACGGCGCGTGCAGGGCCAGACTGGAGGTGGCAACGCCAATCAGCAACGACTGTTTGCCCGCCAGTTGTTGTGCCACGCGGTTGGGAATGTAATTCAGCTCCGCCATCGCCGCTTCCACTTTTTCCCGCGTTTTCGCAGAAACGTGGCTGGCCTGGTTCACCACGCGGGAAACGGTCTGATAAGAGACACCGGCATACTCTGCGACATCGTATAACGTTACTGGTTTCACATTCACCACCCTGAATTGACTCTCTTCCGGGCGCTATCATGCCATACCGCGAAAGGTTTTGCGCCATTCGATGGTGTCCGGGATCTCGACGCTCTCCCTTATGCGACTCCTGCATTAGGAAGCAGCCCAGTAGTAGGTTGAGGCCGTTGAGCACCGCCGCCGCAAGGAATGGTGCATGCCGGCATGCCGCCCTTTCGTCTTCAAGAATTAATTCCCAATTCCCCAGGCATCAAATAAAACGAAAGGCTCAGTCGAAAGACTGGGCCTTTCGTTTTATCTGTTGTTTGTCGGTGAACGCTCTCCTGAGTAGGACAAATCCGCCGGGAGCGGATTTGAACGTTGCGAAGCAACGGCCCGGAGGGTGGCGGGCAGGACGCCCGCCATAAACTGCCAGGAATTAATTCCCCAGGCATCAAATAAAACGAAAGGCTCAGTCGAAAGACTGGGCCTTTCGTTTTATCTGTTGTTTGTCGGTGAACGCTCTCCTGAGTAGGACAAATCCGCCGGGAGCGGATTTGAACGTTGCGAAGCAACGGCCCGGAGGGTGGCGGGCAGGACGCCCGCCATAAACTGCCAGGAATTAATTCCCCAGGCATCAAATAAAACGAAAGGCTCAGTCGAAAGACTGGGCCTTTCGTTTTATCTGTTGTTTGTCGGTGAACGCTCTCCTGAGTAGGACAAATCCGCCGGGAGCGGATTTGAACGTTGCGAAGCAACGGCCCGGAGGGTGGCGGGCAGGACGCCCGCCATAAACTGCCAGGAATTAATTCCCCAGGCATCAAATAAAACGAAAGGCTCAGTCGAAAGACTGGGCCTTTCGTTTTATCTGTTGTTTGTCGGTGAACGCTCTCCTGAGTAGGACAAATCCGCCGGGAGCGGATTTGAACGTTGCGAAGCAACGGCCCGGAGGGTGGCGGGCAGGACGCCCGCCATAAACTGCCAGGAATTAATTCCCCAGGCATCAAATAAAACGAAAGGCTCAGTCGAAAGACTGGGCCTTTCGTTTTATCTGTTGTTTGTCGGTGAACGCTCTCCTGAGTAGGACAAATCCGCCGGGAGCGGATTTGAACGTTGCGAAGCAACGGCCCGGAGGGTGGCGGGCAGGACGCCCGCCATAAACTGCCAGGAATTGGGGATCGGAATTAATTCCCGGTTTAAACCGGGGATCTCGATCCCGCGAAATTAATACGACTCACTATAGGGGAATTGTGAGCGGATAACAATTCCCCTCTAGAAATAATTTTGTTTAACTTTAAGAAGGAGATATACAT**ATGGCTAGCTCGCGAGTCGACGGCGGCCGCGAATTCCTCGAGGGCTCTTC*CTGC*ATCACGGGAGATGCACTAGTTGCCCTACCCGAGGGCGAGTCGGTACGCATCGCCGACATCGTGCCGGGTGCGCGGCCCAACAGTGACAACGCCATCGACCTGAAAGTCCTTGACCGGCATGGCAATCCCGTGCTCGCCGACCGGCTGTTCCACTCCGGCGAGCATCCGGTGTACACGGTGCGTACGGTCGAAGGTCTGCGTGTGACGGGCACCGCGAACCACCCGTTGTTGTGTTTGGTCGACGTCGCCGGGGTGCCGACCCTGCTGTGGAAGCTGATCGACGAAATCAAGCCGGGCGATTACGCGGTGATTCAACGCAGCGCATTCAGCGTCGACTGTGCAGGTTTTGCCCGCGGGAAACCCGAATTTGCGCCCACAACCTACACAGTCGGCGTCCCTGGACTGGTGCGTTTCTTGGAAGCACACCACCGAGACCCGGACGCCCAAGCTATCGCCGACGAGCTGACCGACGGGCGGTTCTACTACGCGAAAGTCGCCAGTGTCACCGACGCCGGCGTGCAGCCGGTGTATAGCCTTCGTGTCGACACGGCAGACCACGCGTTTATCACGAACGGGTTCGTCAGCCACGCTACTGGCCTCACCGGTCTGAACTCAGGCCTCACGACAAATCCTGGTGTATCCGCTTGGCAGGTCAACACAGCTTATACTGCGGGACAATTGGTCACATATAACGGCAAGACGTATAAATGTTTGCAGCCCCACACCTCCTTGGCAGGATGGGAACCATCCAACGTTCCTGCCTTGTGGCAGCTTCAACATCATCATCATCATCACTGA**CTGCAGGAAGGGGATCCGGCTGCTAACAAAGCCCGAAAGGAAGCTGAGTTGGCTGCTGCCACCGCTGAGCAATAACTAGCATAACCCCTTGGGGCCTCTAAACGGGTCTTGAGGGGTTTTTTGCTGAAAGGAGGAACTATATCCGGAT

**His_6_-SUMO sequence in pET-SUMO (red = His_6_; no stop codon included):**

ATGGGCAGCAGCCATCATCATCATCATCACGGCAGCGGCCTGGTGCCGCGCGGCAGCGCTAGCATGTCGGACTCAGAAGTCAATCAAGAAGCTAAGCCAGAGGTCAAGCCAGAAGTCAAGCCTGAGACTCACATCAATTTAAAGGTGTCCGATGGATCTTCAGAGATCTTCTTCAAGATCAAAAAGACCACTCCTTTAAGAAGGCTGATGGAAGCGTTCGCTAAAAGACAGGGTAAGGAAATGGACTCCTTAAGATTCTTGTACGACGGTATTAGAATTCAAGCTGATCAGACCCCTGAAGATTTGGACATGGAGGATAACGATATTATTGAGGCTCACAGAGAACAGATTGGTGGT

**pSPIH6 (*Nde*I, *Pac*I, *Pst*I highlighted, intein sequence in bold and His_6_ underlined,** His_6_-SUMO underlined**, *intein catalytic Cys*):**

AACTACGTCAGGTGGCACTTTTCGGGGAAATGTGCGCGGAACCCCTATTTGTTTATTTTTCTAAATACATTCAAATATGTATCCGCTCATGAGACAATAACCCTGATAAATGCTTCAATAATATTGAAAAAGGAAGAGTATGAGTATTCAACATTTCCGTGTCGCCCTTATTCCCTTTTTTGCGGCATTTTGCCTTCCTGTTTTTGCTCACCCAGAAACGCTGGTGAAAGTAAAAGATGCTGAAGATCAGTTGGGTGCACGAGTGGGTTACATCGAACTGGATCTCAACAGCGGTAAGATCCTTGAGAGTTTTCGCCCCGAAGAACGTTTCCCAATGATGAGCACTTTTAAAGTTCTGCTATGTGGCGCGGTATTATCCCGTGTTGACGCCGGGCAAGAGCAACTCGGTCGCCGCATACACTATTCTCAGAATGACTTGGTTGAGTACTCACCAGTCACAGAAAAGCATCTTACGGATGGCATGACAGTAAGAGAATTATGCAGTGCTGCCATAACCATGAGTGATAACACTGCGGCCAACTTACTTCTGACAACGATCGGAGGACCGAAGGAGCTAACCGCTTTTTTGCACAACATGGGGGATCATGTAACTCGCCTTGATCGTTGGGAACCGGAGCTGAATGAAGCCATACCAAACGACGAGCGTGACACCACGATGCCTGTAGCAATGGCAACAACGTTGCGCAAACTATTAACTGGCGAACTACTTACTCTAGCTTCCCGGCAACAATTAATAGACTGGATGGAGGCGGATAAAGTTGCAGGACCACTTCTGCGCTCGGCCCTTCCGGCTGGCTGGTTTATTGCTGATAAATCTGGAGCCGGTGAGCGTGGGTCTCGCGGTATCATTGCAGCACTGGGGCCAGATGGTAAGCCCTCCCGTATCGTAGTTATCTACACGACGGGGAGTCAGGCAACTATGGATGAACGAAATAGACAGATCGCTGAGATAGGTGCCTCACTGATTAAGCATTGGTAACTGTCAGACCAAGTTTACTCATATATACTTTAGATTGATTTACCCCGGTTGATAATCAGAAAAGCCCCAAAAACAGGAAGATTGTATAAGCAAATATTTAAATTGTAAACGTTAATATTTTGTTAAAATTCGCGTTAAATTTTTGTTAAATCAGCTCATTTTTTAACCAATAGGCCGAAATCGGCAAAATCCCTTATAAATCAAAAGAATAGCCCGAGATAGGGTTGAGTGTTGTTCCAGTTTGGAACAAGAGTCCACTATTAAAGAACGTGGACTCCAACGTCAAAGGGCGAAAAACCGTCTATCAGGGCGATGGCCCACTACGTGAACCATCACCCAAATCAAGTTTTTTGGGGTCGAGGTGCCGTAAAGCACTAAATCGGAACCCTAAAGGGAGCCCCCGATTTAGAGCTTGACGGGGAAAGCCGGCGAACGTGGCGAGAAAGGAAGGGAAGAAAGCGAAAGGAGCGGGCGCTAGGGCGCTGGCAAGTGTAGCGGTCACGCTGCGCGTAACCACCACACCCGCCGCGCTTAATGCGCCGCTACAGGGCGCGTAAAAGGATCTAGGTGAAGATCCTTTTTGATAATCTCATGACCAAAATCCCTTAACGTGAGTTTTCGTTCCACTGAGCGTCAGACCCCGTAGAAAAGATCAAAGGATCTTCTTGAGATCCTTTTTTTCTGCGCGTAATCTGCTGCTTGCAAACAAAAAAACCACCGCTACCAGCGGTGGTTTGTTTGCCGGATCAAGAGCTACCAACTCTTTTTCCGAAGGTAACTGGCTTCAGCAGAGCGCAGATACCAAATACTGTCCTTCTAGTGTAGCCGTAGTTAGGCCACCACTTCAAGAACTCTGTAGCACCGCCTACATACCTCGCTCTGCTAATCCTGTTACCAGTGGCTGCTGCCAGTGGCGATAAGTCGTGTCTTACCGGGTTGGACTCAAGACGATAGTTACCGGATAAGGCGCAGCGGTCGGGCTGAACGGGGGGTTCGTGCACACAGCCCAGCTTGGAGCGAACGACCTACACCGAACTGAGATACCTACAGCGTGAGCTATGAGAAAGCGCCACGCTTCCCGAAGGGAGAAAGGCGGACAGGTATCCGGTAAGCGGCAGGGTCGGAACAGGAGAGCGCACGAGGGAGCTTCCAGGGGGAAACGCCTGGTATCTTTATAGTCCTGTCGGGTTTCGCCACCTCTGACTTGAGCGTCGATTTTTGTGATGCTCGTCAGGGGGGCGGAGCCTATGGAAAAACGCCAGCAACGCGGCCTTTTTACGGTTCCTGGCCTTTTGCTGGCCTTTTGCTCACATGTTCTTTCCTGCGTTATCCCCTGATTCTGTGGATAACCGTATTACCGCCTTTGAGTGAGCTGATACCGCTCGCCGCAGCCGAACGACCGAGCGCAGCGAGTCAGTGAGCGAGGAAGCTATGGTGCACTCTCAGTACAATCTGCTCTGATGCCGCATAGTTAAGCCAGTATACACTCCGCTATCGCTACGTGACTGGGTCATGGCTGCGCCCCGACACCCGCCAACACCCGCTGACGCGCCCTGACGGGCTTGTCTGCTCCCGGCATCCGCTTACAGACAAGCTGTGACCGTCTCCGGGAGCTGCATGTGTCAGAGGTTTTCACCGTCATCACCGAAACGCGCGAGGCAGCTGCGGTAAAGCTCATCAGCGTGGTCGTGCAGCGATTCACAGATGTCTGCCTGTTCATCCGCGTCCAGCTCGTTGAGTTTCTCCAGAAGCGTTAATGTCTGGCTTCTGATAAAGCGGGCCATGTTAAGGGCGGTTTTTTCCTGTTTGGTCACTGATGCCTCCGTGTAAGGGGGATTTCTGTTCATGGGGGTAATGATACCGATGAAACGAGAGAGGATGCTCACGATACGGGTTACTGATGATGAACATGCCCGGTTACTGGAACGTTGTGAGGGTAAACAACTGGCGGTATGGATGCGGCGGGACCAGAGAAAAATCACTCAGGGTCAATGCCAGCCGAACGCCAGCAAGACGTAGCCCAGCGCGTCGGCCGCCATGCCGGCGATAATGGCCTGCTTCTCGCCGAAACGTTTGGTGGCGGGACCAGTGACGAAGGCTTGAGCGAGGGCGTGCAAGATTCCGAATACCGCAAGCGACAGGCCGATCATCGTCGCGCTCCAGCGAAAGCGGTCCTCGCCGAAAATGACCCAGAGCGCTGCCGGCACCTGTCCTACGAGTTGCATGATAAAGAAGACAGTCATAAGTGCGGCGACGATAGTCATGCCCCGCGCCCACCGGAAGGAGCTGACTGGGTTGAAGGCTCTCAAGGGCATCGGTCGAGATCCCGGTGCCTAATGAGTGAGCTAACTTACATTAATTGCGTTGCGCTCACTGCCCGCTTTCCAGTCGGGAAACCTGTCGTGCCAGCTGCATTAATGAATCGGCCAACGCGCGGGGAGAGGCGGTTTGCGTATTGGGCGCCAGGGTGGTTTTTCTTTTCACCAGTGAGACGGGCAACAGCTGATTGCCCTTCACCGCCTGGCCCTGAGAGAGTTGCAGCAAGCGGTCCACGCTGGTTTGCCCCAGCAGGCGAAAATCCTGTTTGATGGTGGTTAACGGCGGGATATAACATGAGCTGTCTTCGGTATCGTCGTATCCCACTACCGAGATATCCGCACCAACGCGCAGCCCGGACTCGGTAATGGCGCGCATTGCGCCCAGCGCCATCTGATCGTTGGCAACCAGCATCGCAGTGGGAACGATGCCCTCATTCAGCATTTGCATGGTTTGTTGAAAACCGGACATGGCACTCCAGTCGCCTTCCCGTTCCGCTATCGGCTGAATTTGATTGCGAGTGAGATATTTATGCCAGCCAGCCAGACGCAGACGCGCCGAGACAGAACTTAATGGGCCCGCTAACAGCGCGATTTGCTGGTGACCCAATGCGACCAGATGCTCCACGCCCAGTCGCGTACCGTCTTCATGGGAGAAAATAATACTGTTGATGGGTGTCTGGTCAGAGACATCAAGAAATAACGCCGGAACATTAGTGCAGGCAGCTTCCACAGCAATGGCATCCTGGTCATCCAGCGGATAGTTAATGATCAGCCCACTGACGCGTTGCGCGAGAAGATTGTGCACCGCCGCTTTACAGGCTTCGACGCCGCTTCGTTCTACCATCGACACCACCACGCTGGCACCCAGTTGATCGGCGCGAGATTTAATCGCCGCGACAATTTGCGACGGCGCGTGCAGGGCCAGACTGGAGGTGGCAACGCCAATCAGCAACGACTGTTTGCCCGCCAGTTGTTGTGCCACGCGGTTGGGAATGTAATTCAGCTCCGCCATCGCCGCTTCCACTTTTTCCCGCGTTTTCGCAGAAACGTGGCTGGCCTGGTTCACCACGCGGGAAACGGTCTGATAAGAGACACCGGCATACTCTGCGACATCGTATAACGTTACTGGTTTCACATTCACCACCCTGAATTGACTCTCTTCCGGGCGCTATCATGCCATACCGCGAAAGGTTTTGCGCCATTCGATGGTGTCCGGGATCTCGACGCTCTCCCTTATGCGACTCCTGCATTAGGAAGCAGCCCAGTAGTAGGTTGAGGCCGTTGAGCACCGCCGCCGCAAGGAATGGTGCATGCCGGCATGCCGCCCTTTCGTCTTCAAGAATTAATTCCCAATTCCCCAGGCATCAAATAAAACGAAAGGCTCAGTCGAAAGACTGGGCCTTTCGTTTTATCTGTTGTTTGTCGGTGAACGCTCTCCTGAGTAGGACAAATCCGCCGGGAGCGGATTTGAACGTTGCGAAGCAACGGCCCGGAGGGTGGCGGGCAGGACGCCCGCCATAAACTGCCAGGAATTAATTCCCCAGGCATCAAATAAAACGAAAGGCTCAGTCGAAAGACTGGGCCTTTCGTTTTATCTGTTGTTTGTCGGTGAACGCTCTCCTGAGTAGGACAAATCCGCCGGGAGCGGATTTGAACGTTGCGAAGCAACGGCCCGGAGGGTGGCGGGCAGGACGCCCGCCATAAACTGCCAGGAATTAATTCCCCAGGCATCAAATAAAACGAAAGGCTCAGTCGAAAGACTGGGCCTTTCGTTTTATCTGTTGTTTGTCGGTGAACGCTCTCCTGAGTAGGACAAATCCGCCGGGAGCGGATTTGAACGTTGCGAAGCAACGGCCCGGAGGGTGGCGGGCAGGACGCCCGCCATAAACTGCCAGGAATTAATTCCCCAGGCATCAAATAAAACGAAAGGCTCAGTCGAAAGACTGGGCCTTTCGTTTTATCTGTTGTTTGTCGGTGAACGCTCTCCTGAGTAGGACAAATCCGCCGGGAGCGGATTTGAACGTTGCGAAGCAACGGCCCGGAGGGTGGCGGGCAGGACGCCCGCCATAAACTGCCAGGAATTAATTCCCCAGGCATCAAATAAAACGAAAGGCTCAGTCGAAAGACTGGGCCTTTCGTTTTATCTGTTGTTTGTCGGTGAACGCTCTCCTGAGTAGGACAAATCCGCCGGGAGCGGATTTGAACGTTGCGAAGCAACGGCCCGGAGGGTGGCGGGCAGGACGCCCGCCATAAACTGCCAGGAATTGGGGATCGGAATTAATTCCCGGTTTAAACCGGGGATCTCGATCCCGCGAAATTAATACGACTCACTATAGGGGAATTGTGAGCGGATAACAATTCCCCTCTAGAAATAATTTTGTTTAACTTTAAGAAGGAGATATACATATGGGCAGCAGCCATCATCATCATCATCACGGCAGCGGCCTGGTGCCGCGCGGCAGCGCTAGCATGTCGGACTCAGAAGTCAATCAAGAAGCTAAGCCAGAGGTCAAGCCAGAAGTCAAGCCTGAGACTCACATCAATTTAAAGGTGTCCGATGGATCTTCAGAGATCTTCTTCAAGATCAAAAAGACCACTCCTTTAAGAAGGCTGATGGAAGCGTTCGCTAAAAGACAGGGTAAGGAAATGGACTCCTTAAGATTCTTGTACGACGGTATTAGAATTCAAGCTGATCAGACCCCTGAAGATTTGGACATGGAGGATAACGATATTATTGAGGCTCACAGAGAACAGATTGGTGGTTTAATTAA***TGC*ATCACGGGAGATGCACTAGTTGCCCTACCCGAGGGCGAGTCGGTACGCATCGCCGACATCGTGCCGGGTGCGCGGCCCAACAGTGACAACGCCATCGACCTGAAAGTCCTTGACCGGCATGGCAATCCCGTGCTCGCCGACCGGCTGTTCCACTCCGGCGAGCATCCGGTGTACACGGTGCGTACGGTCGAAGGTCTGCGTGTGACGGGCACCGCGAACCACCCGTTGTTGTGTTTGGTCGACGTCGCCGGGGTGCCGACCCTGCTGTGGAAGCTGATCGACGAAATCAAGCCGGGCGATTACGCGGTGATTCAACGCAGCGCATTCAGCGTCGACTGTGCAGGTTTTGCCCGCGGGAAACCCGAATTTGCGCCCACAACCTACACAGTCGGCGTCCCTGGACTGGTGCGTTTCTTGGAAGCACACCACCGAGACCCGGACGCCCAAGCTATCGCCGACGAGCTGACCGACGGGCGGTTCTACTACGCGAAAGTCGCCAGTGTCACCGACGCCGGCGTGCAGCCGGTGTATAGCCTTCGTGTCGACACGGCAGACCACGCGTTTATCACGAACGGGTTCGTCAGCCACGCTACTGGCCTCACCGGTCTGAACTCAGGCCTCACGACAAATCCTGGTGTATCCGCTTGGCAGGTCAACACAGCTTATACTGCGGGACAATTGGTCACATATAACGGCAAGACGTATAAATGTTTGCAGCCCCACACCTCCTTGGCAGGATGGGAACCATCCAACGTTCCTGCCTTGTGGCAGCTTCAACATCATCATCATCATCACTGA**CTGCAGGAAGGGGATCCGGCTGCTAACAAAGCCCGAAAGGAAGCTGAGTTGGCTGCTGCCACCGCTGAGCAATAACTAGCATAACCCCTTGGGGCCTCTAAACGGGTCTTGAGGGGTTTTTTGCTGAAAGGAGGAACTATATCCGGAT

**LeuA DNA sequence:**

AAATATTATGGCAACGGTGTGCATTGCACCAAAAGCGGCTGCAGCGTGAACTGGGGCGAAGCGTTTAGCGCGGGCGTGCATCGCCTGGCGAACGGTGGCAACGGCTTTTGG

**pSPIH6-LeuA (His_6_-SUMO underlined, LeuA in orange, intein sequence in bold and His_6_ underlined, *intein catalytic Cys*):**

AACTACGTCAGGTGGCACTTTTCGGGGAAATGTGCGCGGAACCCCTATTTGTTTATTTTTCTAAATACATTCAAATATGTATCCGCTCATGAGACAATAACCCTGATAAATGCTTCAATAATATTGAAAAAGGAAGAGTATGAGTATTCAACATTTCCGTGTCGCCCTTATTCCCTTTTTTGCGGCATTTTGCCTTCCTGTTTTTGCTCACCCAGAAACGCTGGTGAAAGTAAAAGATGCTGAAGATCAGTTGGGTGCACGAGTGGGTTACATCGAACTGGATCTCAACAGCGGTAAGATCCTTGAGAGTTTTCGCCCCGAAGAACGTTCTCCAATGATGAGCACTTTTAAAGTTCTGCTATGTGGCGCGGTATTATCCCGTGTTGACGCCGGGCAAGAGCAACTCGGTCGCCGCATACACTATTCTCAGAATGACTTGGTTGAGTACTCACCAGTCACAGAAAAGCATCTTACGGATGGCATGACAGTAAGAGAATTATGCAGTGCTGCCATAACCATGAGTGATAACACTGCGGCCAACTTACTTCTGACAACGATCGGAGGACCGAAGGAGCTAACCGCTTTTTTGCACAACATGGGGGATCATGTAACTCGCCTTGATCGTTGGGAACCGGAGCTGAATGAAGCCATACCAAACGACGAGCGTGACACCACGATGCCTGTAGCAATGGCAACAACGTTGCGCAAACTATTAACTGGCGAACTACTTACTCTAGCTTCCCGGCAACAATTAATAGACTGGATGGAGGCGGATAAAGTTGCAGGACCACTTCTGCGCTCGGCCCTTCCGGCTGGCTGGTTTATTGCTGATAAATCTGGAGCCGGTGAGCGTGGGTCTCGCGGTATCATTGCAGCACTGGGGCCAGATGGTAAGCCCTCCCGTATCGTAGTTATCTACACGACGGGGAGTCAGGCAACTATGGATGAACGAAATAGACAGATCGCTGAGATAGGTGCCTCACTGATTAAGCATTGGTAACTGTCAGACCAAGTTTACTCATATATACTTTAGATTGATTTACCCCGGTTGATAATCAGAAAAGCCCCAAAAACAGGAAGATTGTATAAGCAAATATTTAAATTGTAAACGTTAATATTTTGTTAAAATTCGCGTTAAATTTTTGTTAAATCAGCTCATTTTTTAACCAATAGGCCGAAATCGGCAAAATCCCTTATAAATCAAAAGAATAGCCCGAGATAGGGTTGAGTGTTGTTCCAGTTTGGAACAAGAGTCCACTATTAAAGAACGTGGACTCCAACGTCAAAGGGCGAAAAACCGTCTATCAGGGCGATGGCCCACTACGTGAACCATCACCCAAATCAAGTTTTTTGGGGTCGAGGTGCCGTAAAGCACTAAATCGGAACCCTAAAGGGAGCCCCCGATTTAGAGCTTGACGGGGAAAGCCGGCGAACGTGGCGAGAAAGGAAGGGAAGAAAGCGAAAGGAGCGGGCGCTAGGGCGCTGGCAAGTGTAGCGGTCACGCTGCGCGTAACCACCACACCCGCCGCGCTTAATGCGCCGCTACAGGGCGCGTAAAAGGATCTAGGTGAAGATCCTTTTTGATAATCTCATGACCAAAATCCCTTAACGTGAGTTTTCGTTCCACTGAGCGTCAGACCCCGTAGAAAAGATCAAAGGATCTTCTTGAGATCCTTTTTTTCTGCGCGTAATCTGCTGCTTGCAAACAAAAAAACCACCGCTACCAGCGGTGGTTTGTTTGCCGGATCAAGAGCTACCAACTCTTTTTCCGAAGGTAACTGGCTTCAGCAGAGCGCAGATACCAAATACTGTCCTTCTAGTGTAGCCGTAGTTAGGCCACCACTTCAAGAACTCTGTAGCACCGCCTACATACCTCGCTCTGCTAATCCTGTTACCAGTGGCTGCTGCCAGTGGCGATAAGTCGTGTCTTACCGGGTTGGACTCAAGACGATAGTTACCGGATAAGGCGCAGCGGTCGGGCTGAACGGGGGGTTCGTGCACACAGCCCAGCTTGGAGCGAACGACCTACACCGAACTGAGATACCTACAGCGTGAGCTATGAGAAAGCGCCACGCTTCCCGAAGGGAGAAAGGCGGACAGGTATCCGGTAAGCGGCAGGGTCGGAACAGGAGAGCGCACGAGGGAGCTTCCAGGGGGAAACGCCTGGTATCTTTATAGTCCTGTCGGGTTTCGCCACCTCTGACTTGAGCGTCGATTTTTGTGATGCTCGTCAGGGGGGCGGAGCCTATGGAAAAACGCCAGCAACGCGGCCTTTTTACGGTTCCTGGCCTTTTGCTGGCCTTTTGCTCACATGTTCTTTCCTGCGTTATCCCCTGATTCTGTGGATAACCGTATTACCGCCTTTGAGTGAGCTGATACCGCTCGCCGCAGCCGAACGACCGAGCGCAGCGAGTCAGTGAGCGAGGAAGCTATGGTGCACTCTCAGTACAATCTGCTCTGATGCCGCATAGTTAAGCCAGTATACACTCCGCTATCGCTACGTGACTGGGTCATGGCTGCGCCCCGACACCCGCCAACACCCGCTGACGCGCCCTGACGGGCTTGTCTGCTCCCGGCATCCGCTTACAGACAAGCTGTGACCGTCTCCGGGAGCTGCATGTGTCAGAGGTTTTCACCGTCATCACCGAAACGCGCGAGGCAGCTGCGGTAAAGCTCATCAGCGTGGTCGTGCAGCGATTCACAGATGTCTGCCTGTTCATCCGCGTCCAGCTCGTTGAGTTTCTCCAGAAGCGTTAATGTCTGGCTTCTGATAAAGCGGGCCATGTTAAGGGCGGTTTTTTCCTGTTTGGTCACTGATGCCTCCGTGTAAGGGGGATTTCTGTTCATGGGGGTAATGATACCGATGAAACGAGAGAGGATGCTCACGATACGGGTTACTGATGATGAACATGCCCGGTTACTGGAACGTTGTGAGGGTAAACAACTGGCGGTATGGATGCGGCGGGACCAGAGAAAAATCACTCAGGGTCAATGCCAGCCGAACGCCAGCAAGACGTAGCCCAGCGCGTCGGCCGCCATGCCGGCGATAATGGCCTGCTTCTCGCCGAAACGTTTGGTGGCGGGACCAGTGACGAAGGCTTGAGCGAGGGCGTGCAAGATTCCGAATACCGCAAGCGACAGGCCGATCATCGTCGCGCTCCAGCGAAAGCGGTCCTCGCCGAAAATGACCCAGAGCGCTGCCGGCACCTGTCCTACGAGTTGCATGATAAAGAAGACAGTCATAAGTGCGGCGACGATAGTCATGCCCCGCGCCCACCGGAAGGAGCTGACTGGGTTGAAGGCTCTCAAGGGCATCGGTCGAGATCCCGGTGCCTAATGAGTGAGCTAACTTACATTAATTGCGTTGCGCTCACTGCCCGCTTTCCAGTCGGGAAACCTGTCGTGCCAGCTGCATTAATGAATCGGCCAACGCGCGGGGAGAGGCGGTTTGCGTATTGGGCGCCAGGGTGGTTTTTCTTTTCACCAGTGAGACGGGCAACAGCTGATTGCCCTTCACCGCCTGGCCCTGAGAGAGTTGCAGCAAGCGGTCCACGCTGGTTTGCCCCAGCAGGCGAAAATCCTGTTTGATGGTGGTTAACGGCGGGATATAACATGAGCTGTCTTCGGTATCGTCGTATCCCACTACCGAGATATCCGCACCAACGCGCAGCCCGGACTCGGTAATGGCGCGCATTGCGCCCAGCGCCATCTGATCGTTGGCAACCAGCATCGCAGTGGGAACGATGCCCTCATTCAGCATTTGCATGGTTTGTTGAAAACCGGACATGGCACTCCAGTCGCCTTCCCGTTCCGCTATCGGCTGAATTTGATTGCGAGTGAGATATTTATGCCAGCCAGCCAGACGCAGACGCGCCGAGACAGAACTTAATGGGCCCGCTAACAGCGCGATTTGCTGGTGACCCAATGCGACCAGATGCTCCACGCCCAGTCGCGTACCGTCTTCATGGGAGAAAATAATACTGTTGATGGGTGTCTGGTCAGAGACATCAAGAAATAACGCCGGAACATTAGTGCAGGCAGCTTCCACAGCAATGGCATCCTGGTCATCCAGCGGATAGTTAATGATCAGCCCACTGACGCGTTGCGCGAGAAGATTGTGCACCGCCGCTTTACAGGCTTCGACGCCGCTTCGTTCTACCATCGACACCACCACGCTGGCACCCAGTTGATCGGCGCGAGATTTAATCGCCGCGACAATTTGCGACGGCGCGTGCAGGGCCAGACTGGAGGTGGCAACGCCAATCAGCAACGACTGTTTGCCCGCCAGTTGTTGTGCCACGCGGTTGGGAATGTAATTCAGCTCCGCCATCGCCGCTTCCACTTTTTCCCGCGTTTTCGCAGAAACGTGGCTGGCCTGGTTCACCACGCGGGAAACGGTCTGATAAGAGACACCGGCATACTCTGCGACATCGTATAACGTTACTGGTTTCACATTCACCACCCTGAATTGACTCTCTTCCGGGCGCTATCATGCCATACCGCGAAAGGTTTTGCGCCATTCGATGGTGTCCGGGATCTCGACGCTCTCCCTTATGCGACTCCTGCATTAGGAAGCAGCCCAGTAGTAGGTTGAGGCCGTTGAGCACCGCCGCCGCAAGGAATGGTGCATGCCGGCATGCCGCCCTTTCGTCTTCAAGAATTAATTCCCAATTCCCCAGGCATCAAATAAAACGAAAGGCTCAGTCGAAAGACTGGGCCTTTCGTTTTATCTGTTGTTTGTCGGTGAACGCTCTCCTGAGTAGGACAAATCCGCCGGGAGCGGATTTGAACGTTGCGAAGCAACGGCCCGGAGGGTGGCGGGCAGGACGCCCGCCATAAACTGCCAGGAATTAATTCCCCAGGCATCAAATAAAACGAAAGGCTCAGTCGAAAGACTGGGCCTTTCGTTTTATCTGTTGTTTGTCGGTGAACGCTCTCCTGAGTAGGACAAATCCGCCGGGAGCGGATTTGAACGTTGCGAAGCAACGGCCCGGAGGGTGGCGGGCAGGACGCCCGCCATAAACTGCCAGGAATTAATTCCCCAGGCATCAAATAAAACGAAAGGCTCAGTCGAAAGACTGGGCCTTTCGTTTTATCTGTTGTTTGTCGGTGAACGCTCTCCTGAGTAGGACAAATCCGCCGGGAGCGGATTTGAACGTTGCGAAGCAACGGCCCGGAGGGTGGCGGGCAGGACGCCCGCCATAAACTGCCAGGAATTAATTCCCCAGGCATCAAATAAAACGAAAGGCTCAGTCGAAAGACTGGGCCTTTCGTTTTATCTGTTGTTTGTCGGTGAACGCTCTCCTGAGTAGGACAAATCCGCCGGGAGCGGATTTGAACGTTGCGAAGCAACGGCCCGGAGGGTGGCGGGCAGGACGCCCGCCATAAACTGCCAGGAATTAATTCCCCAGGCATCAAATAAAACGAAAGGCTCAGTCGAAAGACTGGGCCTTTCGTTTTATCTGTTGTTTGTCGGTGAACGCTCTCCTGAGTAGGACAAATCCGCCGGGAGCGGATTTGAACGTTGCGAAGCAACGGCCCGGAGGGTGGCGGGCAGGACGCCCGCCATAAACTGCCAGGAATTGGGGATCGGAATTAATTCCCGGTTTAAACCGGGGATCTCGATCCCGCGAAATTAATACGACTCACTATAGGGGAATTGTGAGCGGATAACAATTCCCCTCTAGAAATAATTTTGTTTAACTTTAAGAAGGAGATATACATATGGGCAGCAGCCATCATCATCATCATCACGGCAGCGGCCTGGTGCCGCGCGGCAGCGCTAGCATGTCGGACTCAGAAGTCAATCAAGAAGCTAAGCCAGAGGTCAAGCCAGAAGTCAAGCCTGAGACTCACATCAATTTAAAGGTGTCCGATGGATCTTCAGAGATCTTCTTCAAGATCAAAAAGACCACTCCTTTAAGAAGGCTGATGGAAGCGTTCGCTAAAAGACAGGGTAAGGAAATGGACTCCTTAAGATTCTTGTACGACGGTATTAGAATTCAAGCTGATCAGACCCCTGAAGATTTGGACATGGAGGATAACGATATTATTGAGGCTCACAGAGAACAGATTGGTGGTAAATATTATGGCAACGGTGTGCATTGCACCAAAAGCGGCTGCAGCGTGAACTGGGGCGAAGCGTTTAGCGCGGGCGTGCATCGCCTGGCGAACGGTGGCAACGGCTTTTGG***TGC*ATCACGGGAGATGCACTAGTTGCCCTACCCGAGGGCGAGTCGGTACGCATCGCCGACATCGTGCCGGGTGCGCGGCCCAACAGTGACAACGCCATCGACCTGAAAGTCCTTGACCGGCATGGCAATCCCGTGCTCGCCGACCGGCTGTTCCACTCCGGCGAGCATCCGGTGTACACGGTGCGTACGGTCGAAGGTCTGCGTGTGACGGGCACCGCGAACCACCCGTTGTTGTGTTTGGTCGACGTCGCCGGGGTGCCGACCCTGCTGTGGAAGCTGATCGACGAAATCAAGCCGGGCGATTACGCGGTGATTCAACGCAGCGCATTCAGCGTCGACTGTGCAGGTTTTGCCCGCGGGAAACCCGAATTTGCGCCCACAACCTACACAGTCGGCGTCCCTGGACTGGTGCGTTTCTTGGAAGCACACCACCGAGACCCGGACGCCCAAGCTATCGCCGACGAGCTGACCGACGGGCGGTTCTACTACGCGAAAGTCGCCAGTGTCACCGACGCCGGCGTGCAGCCGGTGTATAGCCTTCGTGTCGACACGGCAGACCACGCGTTTATCACGAACGGGTTCGTCAGCCACGCTACTGGCCTCACCGGTCTGAACTCAGGCCTCACGACAAATCCTGGTGTATCCGCTTGGCAGGTCAACACAGCTTATACTGCGGGACAATTGGTCACATATAACGGCAAGACGTATAAATGTTTGCAGCCCCACACCTCCTTGGCAGGATGGGAACCATCCAACGTTCCTGCCTTGTGGCAGCTTCAACATCATCATCATCATCACTGA**CTGCAGGAAGGGGATCCGGCTGCTAACAAAGCCCGAAAGGAAGCTGAGTTGGCTGCTGCCACCGCTGAGCAATAACTAGCATAACCCCTTGGGGCCTCTAAACGGGTCTTGAGGGGTTTTTTGCTGAAAGGAGGAACTATATCCGGAT

**LcnA DNA sequence:**

AAACTGACCTTTATTCAGAGCACCGCGGCGGGCGATCTGTATTATAACACCAACACCCATAAATATGTGTATCAGCAGACCCAGAACGCGTTTGGCGCGGCGGCGAACACCATTGTGAACGGCTGGATGGGCGGCGCGGCGGGCGGCTTTGGCCTGCATCAT

**pSPIH6-LcnA (His_6_-SUMO underlined, LcnA in red, intein sequence in bold and His_6_ underlined, *intein catalytic Cys*):**

AACTACGTCAGGTGGCACTTTTCGGGGAAATGTGCGCGGAACCCCTATTTGTTTATTTTTCTAAATACATTCAAATATGTATCCGCTCATGAGACAATAACCCTGATAAATGCTTCAATAATATTGAAAAAGGAAGAGTATGAGTATTCAACATTTCCGTGTCGCCCTTATTCCCTTTTTTGCGGCATTTTGCCTTCCTGTTTTTGCTCACCCAGAAACGCTGGTGAAAGTAAAAGATGCTGAAGATCAGTTGGGTGCACGAGTGGGTTACATCGAACTGGATCTCAACAGCGGTAAGATCCTTGAGAGTTTTCGCCCCGAAGAACGTTCTCCAATGATGAGCACTTTTAAAGTTCTGCTATGTGGCGCGGTATTATCCCGTGTTGACGCCGGGCAAGAGCAACTCGGTCGCCGCATACACTATTCTCAGAATGACTTGGTTGAGTACTCACCAGTCACAGAAAAGCATCTTACGGATGGCATGACAGTAAGAGAATTATGCAGTGCTGCCATAACCATGAGTGATAACACTGCGGCCAACTTACTTCTGACAACGATCGGAGGACCGAAGGAGCTAACCGCTTTTTTGCACAACATGGGGGATCATGTAACTCGCCTTGATCGTTGGGAACCGGAGCTGAATGAAGCCATACCAAACGACGAGCGTGACACCACGATGCCTGTAGCAATGGCAACAACGTTGCGCAAACTATTAACTGGCGAACTACTTACTCTAGCTTCCCGGCAACAATTAATAGACTGGATGGAGGCGGATAAAGTTGCAGGACCACTTCTGCGCTCGGCCCTTCCGGCTGGCTGGTTTATTGCTGATAAATCTGGAGCCGGTGAGCGTGGGTCTCGCGGTATCATTGCAGCACTGGGGCCAGATGGTAAGCCCTCCCGTATCGTAGTTATCTACACGACGGGGAGTCAGGCAACTATGGATGAACGAAATAGACAGATCGCTGAGATAGGTGCCTCACTGATTAAGCATTGGTAACTGTCAGACCAAGTTTACTCATATATACTTTAGATTGATTTACCCCGGTTGATAATCAGAAAAGCCCCAAAAACAGGAAGATTGTATAAGCAAATATTTAAATTGTAAACGTTAATATTTTGTTAAAATTCGCGTTAAATTTTTGTTAAATCAGCTCATTTTTTAACCAATAGGCCGAAATCGGCAAAATCCCTTATAAATCAAAAGAATAGCCCGAGATAGGGTTGAGTGTTGTTCCAGTTTGGAACAAGAGTCCACTATTAAAGAACGTGGACTCCAACGTCAAAGGGCGAAAAACCGTCTATCAGGGCGATGGCCCACTACGTGAACCATCACCCAAATCAAGTTTTTTGGGGTCGAGGTGCCGTAAAGCACTAAATCGGAACCCTAAAGGGAGCCCCCGATTTAGAGCTTGACGGGGAAAGCCGGCGAACGTGGCGAGAAAGGAAGGGAAGAAAGCGAAAGGAGCGGGCGCTAGGGCGCTGGCAAGTGTAGCGGTCACGCTGCGCGTAACCACCACACCCGCCGCGCTTAATGCGCCGCTACAGGGCGCGTAAAAGGATCTAGGTGAAGATCCTTTTTGATAATCTCATGACCAAAATCCCTTAACGTGAGTTTTCGTTCCACTGAGCGTCAGACCCCGTAGAAAAGATCAAAGGATCTTCTTGAGATCCTTTTTTTCTGCGCGTAATCTGCTGCTTGCAAACAAAAAAACCACCGCTACCAGCGGTGGTTTGTTTGCCGGATCAAGAGCTACCAACTCTTTTTCCGAAGGTAACTGGCTTCAGCAGAGCGCAGATACCAAATACTGTCCTTCTAGTGTAGCCGTAGTTAGGCCACCACTTCAAGAACTCTGTAGCACCGCCTACATACCTCGCTCTGCTAATCCTGTTACCAGTGGCTGCTGCCAGTGGCGATAAGTCGTGTCTTACCGGGTTGGACTCAAGACGATAGTTACCGGATAAGGCGCAGCGGTCGGGCTGAACGGGGGGTTCGTGCACACAGCCCAGCTTGGAGCGAACGACCTACACCGAACTGAGATACCTACAGCGTGAGCTATGAGAAAGCGCCACGCTTCCCGAAGGGAGAAAGGCGGACAGGTATCCGGTAAGCGGCAGGGTCGGAACAGGAGAGCGCACGAGGGAGCTTCCAGGGGGAAACGCCTGGTATCTTTATAGTCCTGTCGGGTTTCGCCACCTCTGACTTGAGCGTCGATTTTTGTGATGCTCGTCAGGGGGGCGGAGCCTATGGAAAAACGCCAGCAACGCGGCCTTTTTACGGTTCCTGGCCTTTTGCTGGCCTTTTGCTCACATGTTCTTTCCTGCGTTATCCCCTGATTCTGTGGATAACCGTATTACCGCCTTTGAGTGAGCTGATACCGCTCGCCGCAGCCGAACGACCGAGCGCAGCGAGTCAGTGAGCGAGGAAGCTATGGTGCACTCTCAGTACAATCTGCTCTGATGCCGCATAGTTAAGCCAGTATACACTCCGCTATCGCTACGTGACTGGGTCATGGCTGCGCCCCGACACCCGCCAACACCCGCTGACGCGCCCTGACGGGCTTGTCTGCTCCCGGCATCCGCTTACAGACAAGCTGTGACCGTCTCCGGGAGCTGCATGTGTCAGAGGTTTTCACCGTCATCACCGAAACGCGCGAGGCAGCTGCGGTAAAGCTCATCAGCGTGGTCGTGCAGCGATTCACAGATGTCTGCCTGTTCATCCGCGTCCAGCTCGTTGAGTTTCTCCAGAAGCGTTAATGTCTGGCTTCTGATAAAGCGGGCCATGTTAAGGGCGGTTTTTTCCTGTTTGGTCACTGATGCCTCCGTGTAAGGGGGATTTCTGTTCATGGGGGTAATGATACCGATGAAACGAGAGAGGATGCTCACGATACGGGTTACTGATGATGAACATGCCCGGTTACTGGAACGTTGTGAGGGTAAACAACTGGCGGTATGGATGCGGCGGGACCAGAGAAAAATCACTCAGGGTCAATGCCAGCCGAACGCCAGCAAGACGTAGCCCAGCGCGTCGGCCGCCATGCCGGCGATAATGGCCTGCTTCTCGCCGAAACGTTTGGTGGCGGGACCAGTGACGAAGGCTTGAGCGAGGGCGTGCAAGATTCCGAATACCGCAAGCGACAGGCCGATCATCGTCGCGCTCCAGCGAAAGCGGTCCTCGCCGAAAATGACCCAGAGCGCTGCCGGCACCTGTCCTACGAGTTGCATGATAAAGAAGACAGTCATAAGTGCGGCGACGATAGTCATGCCCCGCGCCCACCGGAAGGAGCTGACTGGGTTGAAGGCTCTCAAGGGCATCGGTCGAGATCCCGGTGCCTAATGAGTGAGCTAACTTACATTAATTGCGTTGCGCTCACTGCCCGCTTTCCAGTCGGGAAACCTGTCGTGCCAGCTGCATTAATGAATCGGCCAACGCGCGGGGAGAGGCGGTTTGCGTATTGGGCGCCAGGGTGGTTTTTCTTTTCACCAGTGAGACGGGCAACAGCTGATTGCCCTTCACCGCCTGGCCCTGAGAGAGTTGCAGCAAGCGGTCCACGCTGGTTTGCCCCAGCAGGCGAAAATCCTGTTTGATGGTGGTTAACGGCGGGATATAACATGAGCTGTCTTCGGTATCGTCGTATCCCACTACCGAGATATCCGCACCAACGCGCAGCCCGGACTCGGTAATGGCGCGCATTGCGCCCAGCGCCATCTGATCGTTGGCAACCAGCATCGCAGTGGGAACGATGCCCTCATTCAGCATTTGCATGGTTTGTTGAAAACCGGACATGGCACTCCAGTCGCCTTCCCGTTCCGCTATCGGCTGAATTTGATTGCGAGTGAGATATTTATGCCAGCCAGCCAGACGCAGACGCGCCGAGACAGAACTTAATGGGCCCGCTAACAGCGCGATTTGCTGGTGACCCAATGCGACCAGATGCTCCACGCCCAGTCGCGTACCGTCTTCATGGGAGAAAATAATACTGTTGATGGGTGTCTGGTCAGAGACATCAAGAAATAACGCCGGAACATTAGTGCAGGCAGCTTCCACAGCAATGGCATCCTGGTCATCCAGCGGATAGTTAATGATCAGCCCACTGACGCGTTGCGCGAGAAGATTGTGCACCGCCGCTTTACAGGCTTCGACGCCGCTTCGTTCTACCATCGACACCACCACGCTGGCACCCAGTTGATCGGCGCGAGATTTAATCGCCGCGACAATTTGCGACGGCGCGTGCAGGGCCAGACTGGAGGTGGCAACGCCAATCAGCAACGACTGTTTGCCCGCCAGTTGTTGTGCCACGCGGTTGGGAATGTAATTCAGCTCCGCCATCGCCGCTTCCACTTTTTCCCGCGTTTTCGCAGAAACGTGGCTGGCCTGGTTCACCACGCGGGAAACGGTCTGATAAGAGACACCGGCATACTCTGCGACATCGTATAACGTTACTGGTTTCACATTCACCACCCTGAATTGACTCTCTTCCGGGCGCTATCATGCCATACCGCGAAAGGTTTTGCGCCATTCGATGGTGTCCGGGATCTCGACGCTCTCCCTTATGCGACTCCTGCATTAGGAAGCAGCCCAGTAGTAGGTTGAGGCCGTTGAGCACCGCCGCCGCAAGGAATGGTGCATGCCGGCATGCCGCCCTTTCGTCTTCAAGAATTAATTCCCAATTCCCCAGGCATCAAATAAAACGAAAGGCTCAGTCGAAAGACTGGGCCTTTCGTTTTATCTGTTGTTTGTCGGTGAACGCTCTCCTGAGTAGGACAAATCCGCCGGGAGCGGATTTGAACGTTGCGAAGCAACGGCCCGGAGGGTGGCGGGCAGGACGCCCGCCATAAACTGCCAGGAATTAATTCCCCAGGCATCAAATAAAACGAAAGGCTCAGTCGAAAGACTGGGCCTTTCGTTTTATCTGTTGTTTGTCGGTGAACGCTCTCCTGAGTAGGACAAATCCGCCGGGAGCGGATTTGAACGTTGCGAAGCAACGGCCCGGAGGGTGGCGGGCAGGACGCCCGCCATAAACTGCCAGGAATTAATTCCCCAGGCATCAAATAAAACGAAAGGCTCAGTCGAAAGACTGGGCCTTTCGTTTTATCTGTTGTTTGTCGGTGAACGCTCTCCTGAGTAGGACAAATCCGCCGGGAGCGGATTTGAACGTTGCGAAGCAACGGCCCGGAGGGTGGCGGGCAGGACGCCCGCCATAAACTGCCAGGAATTAATTCCCCAGGCATCAAATAAAACGAAAGGCTCAGTCGAAAGACTGGGCCTTTCGTTTTATCTGTTGTTTGTCGGTGAACGCTCTCCTGAGTAGGACAAATCCGCCGGGAGCGGATTTGAACGTTGCGAAGCAACGGCCCGGAGGGTGGCGGGCAGGACGCCCGCCATAAACTGCCAGGAATTAATTCCCCAGGCATCAAATAAAACGAAAGGCTCAGTCGAAAGACTGGGCCTTTCGTTTTATCTGTTGTTTGTCGGTGAACGCTCTCCTGAGTAGGACAAATCCGCCGGGAGCGGATTTGAACGTTGCGAAGCAACGGCCCGGAGGGTGGCGGGCAGGACGCCCGCCATAAACTGCCAGGAATTGGGGATCGGAATTAATTCCCGGTTTAAACCGGGGATCTCGATCCCGCGAAATTAATACGACTCACTATAGGGGAATTGTGAGCGGATAACAATTCCCCTCTAGAAATAATTTTGTTTAACTTTAAGAAGGAGATATACATATGGGCAGCAGCCATCATCATCATCATCACGGCAGCGGCCTGGTGCCGCGCGGCAGCGCTAGCATGTCGGACTCAGAAGTCAATCAAGAAGCTAAGCCAGAGGTCAAGCCAGAAGTCAAGCCTGAGACTCACATCAATTTAAAGGTGTCCGATGGATCTTCAGAGATCTTCTTCAAGATCAAAAAGACCACTCCTTTAAGAAGGCTGATGGAAGCGTTCGCTAAAAGACAGGGTAAGGAAATGGACTCCTTAAGATTCTTGTACGACGGTATTAGAATTCAAGCTGATCAGACCCCTGAAGATTTGGACATGGAGGATAACGATATTATTGAGGCTCACAGAGAACAGATTGGTGGTAAACTGACCTTTATTCAGAGCACCGCGGCGGGCGATCTGTATTATAACACCAACACCCATAAATATGTGTATCAGCAGACCCAGAACGCGTTTGGCGCGGCGGCGAACACCATTGTGAACGGCTGGATGGGCGGCGCGGCGGGCGGCTTTGGCCTGCATCAT***TGC*ATCACGGGAGATGCACTAGTTGCCCTACCCGAGGGCGAGTCGGTACGCATCGCCGACATCGTGCCGGGTGCGCGGCCCAACAGTGACAACGCCATCGACCTGAAAGTCCTTGACCGGCATGGCAATCCCGTGCTCGCCGACCGGCTGTTCCACTCCGGCGAGCATCCGGTGTACACGGTGCGTACGGTCGAAGGTCTGCGTGTGACGGGCACCGCGAACCACCCGTTGTTGTGTTTGGTCGACGTCGCCGGGGTGCCGACCCTGCTGTGGAAGCTGATCGACGAAATCAAGCCGGGCGATTACGCGGTGATTCAACGCAGCGCATTCAGCGTCGACTGTGCAGGTTTTGCCCGCGGGAAACCCGAATTTGCGCCCACAACCTACACAGTCGGCGTCCCTGGACTGGTGCGTTTCTTGGAAGCACACCACCGAGACCCGGACGCCCAAGCTATCGCCGACGAGCTGACCGACGGGCGGTTCTACTACGCGAAAGTCGCCAGTGTCACCGACGCCGGCGTGCAGCCGGTGTATAGCCTTCGTGTCGACACGGCAGACCACGCGTTTATCACGAACGGGTTCGTCAGCCACGCTACTGGCCTCACCGGTCTGAACTCAGGCCTCACGACAAATCCTGGTGTATCCGCTTGGCAGGTCAACACAGCTTATACTGCGGGACAATTGGTCACATATAACGGCAAGACGTATAAATGTTTGCAGCCCCACACCTCCTTGGCAGGATGGGAACCATCCAACGTTCCTGCCTTGTGGCAGCTTCAACATCATCATCATCATCACTGA**CTGCAGGAAGGGGATCCGGCTGCTAACAAAGCCCGAAAGGAAGCTGAGTTGGCTGCTGCCACCGCTGAGCAATAACTAGCATAACCCCTTGGGGCCTCTAAACGGGTCTTGAGGGGTTTTTTGCTGAAAGGAGGAACTATATCCGGAT

**HPLC Chromatograms:**

**LeuA HPLC chromatogram (elution at 37% solvent B, 15.7 min, HPLC method 1)**

**LcnA HPLC chromatogram (elution at 42% solvent B, 10.2 min, HPLC method 2)**

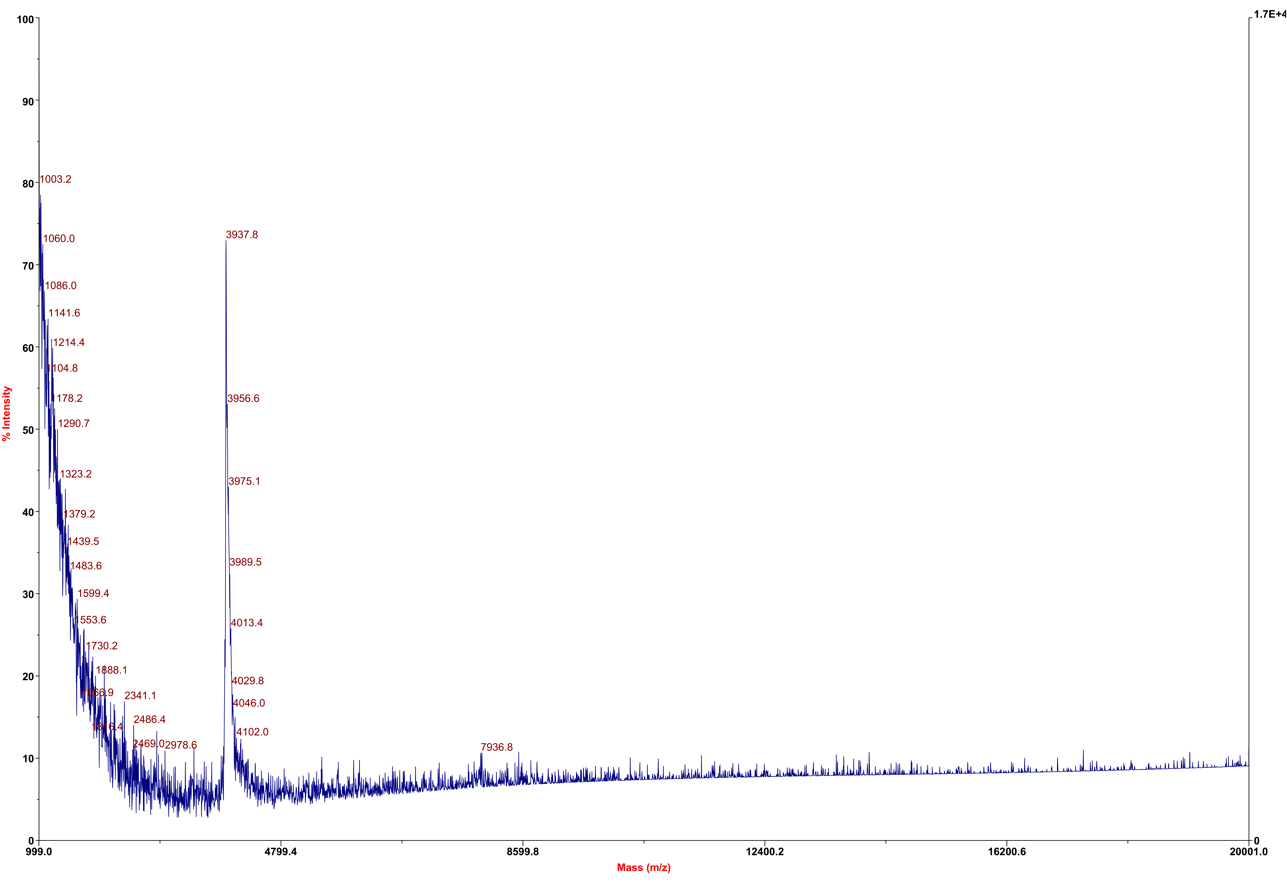
**LeuA raw MALDI-TOF MS:**

**LcnA raw MALDI-TOF MS:**


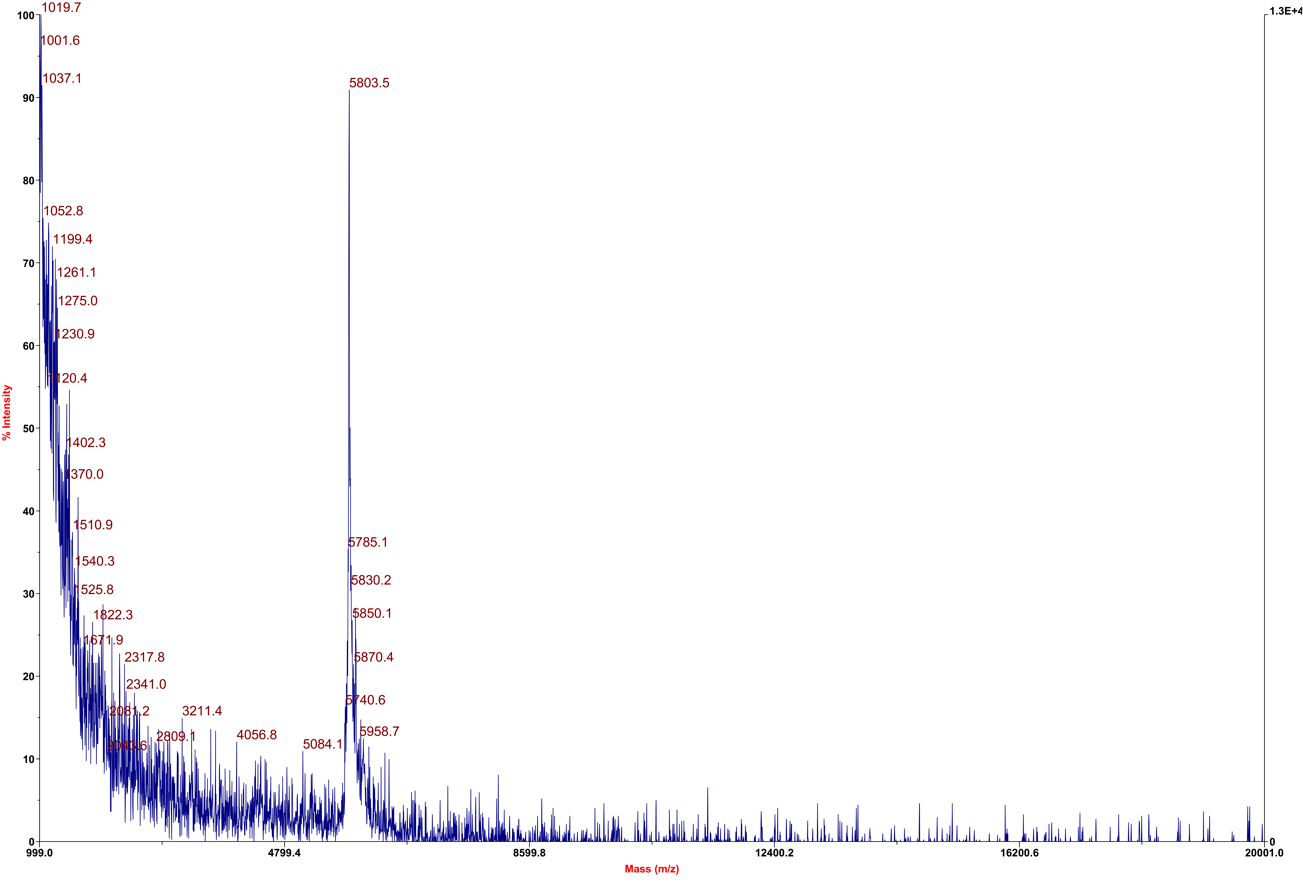


**Unedited SDS-PAGE images:**

**Figure 3:**

**
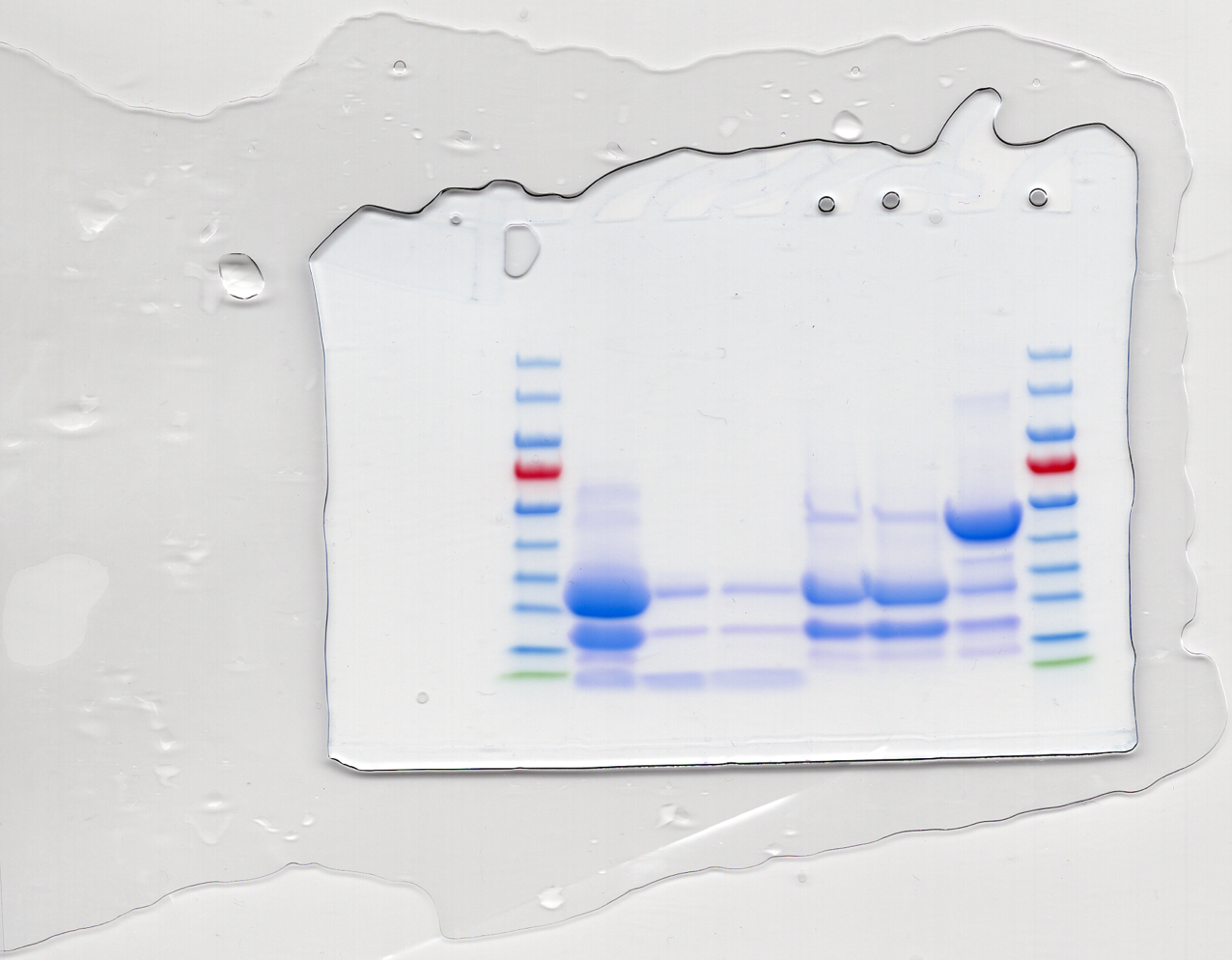
**

**Supplementary figure 2:**

**
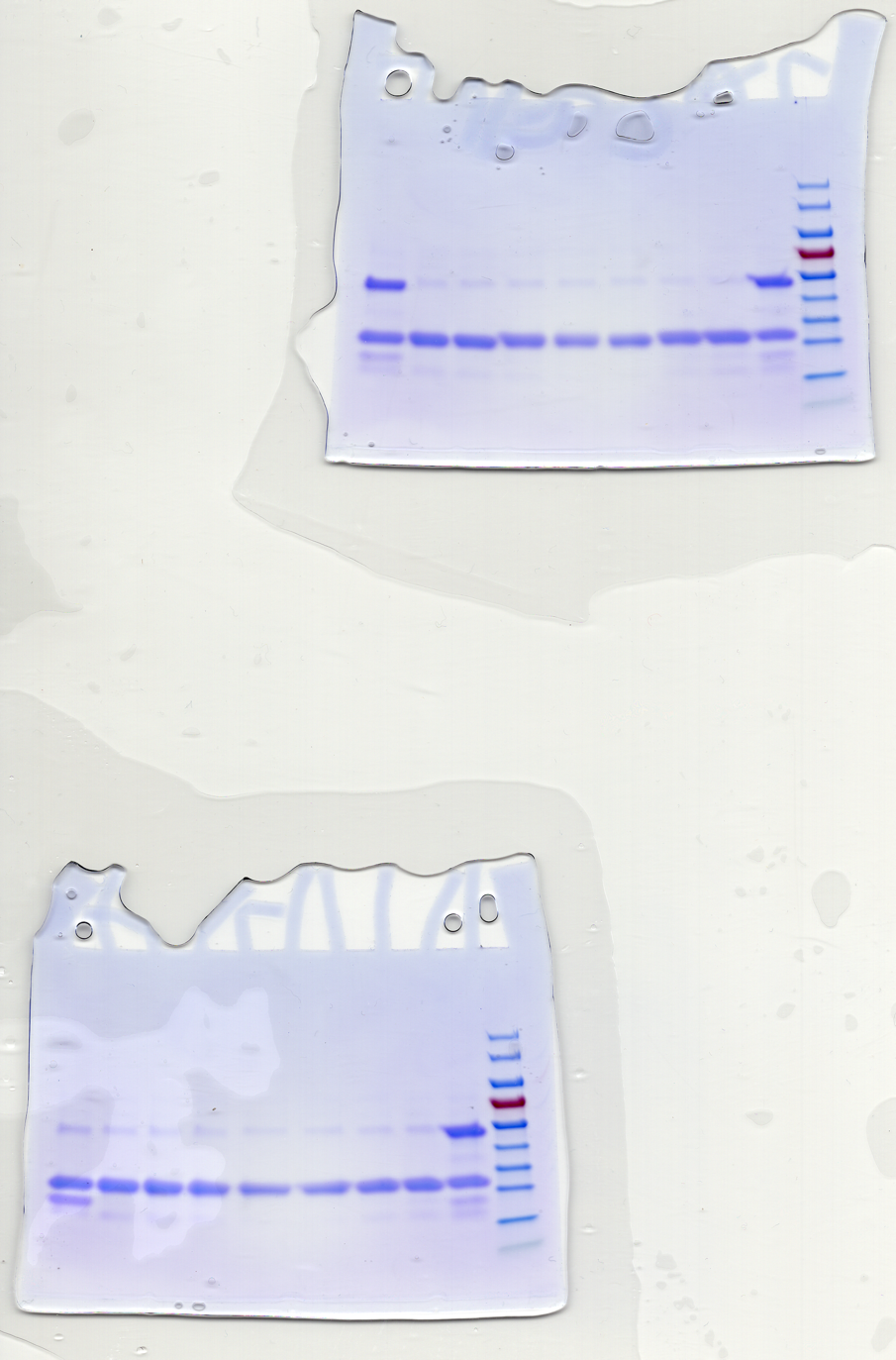
**

**
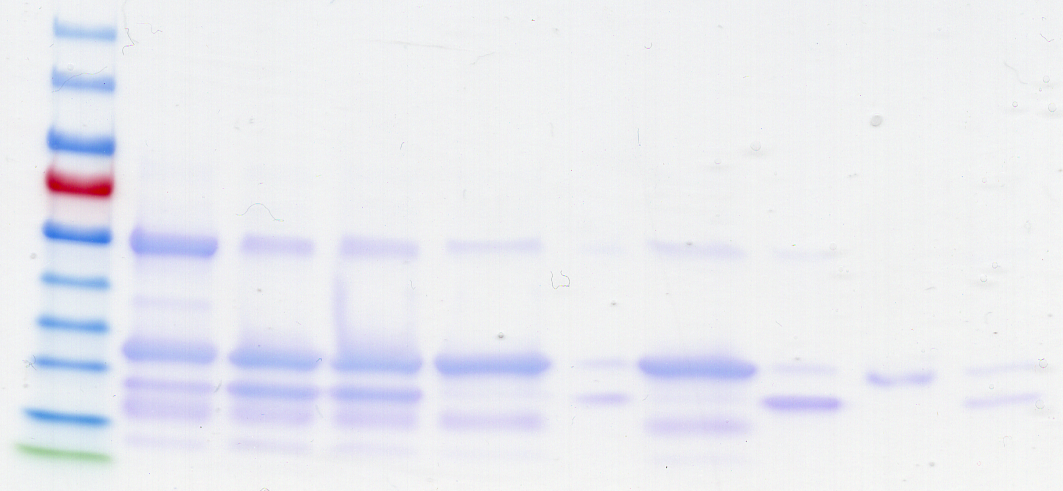
**

**
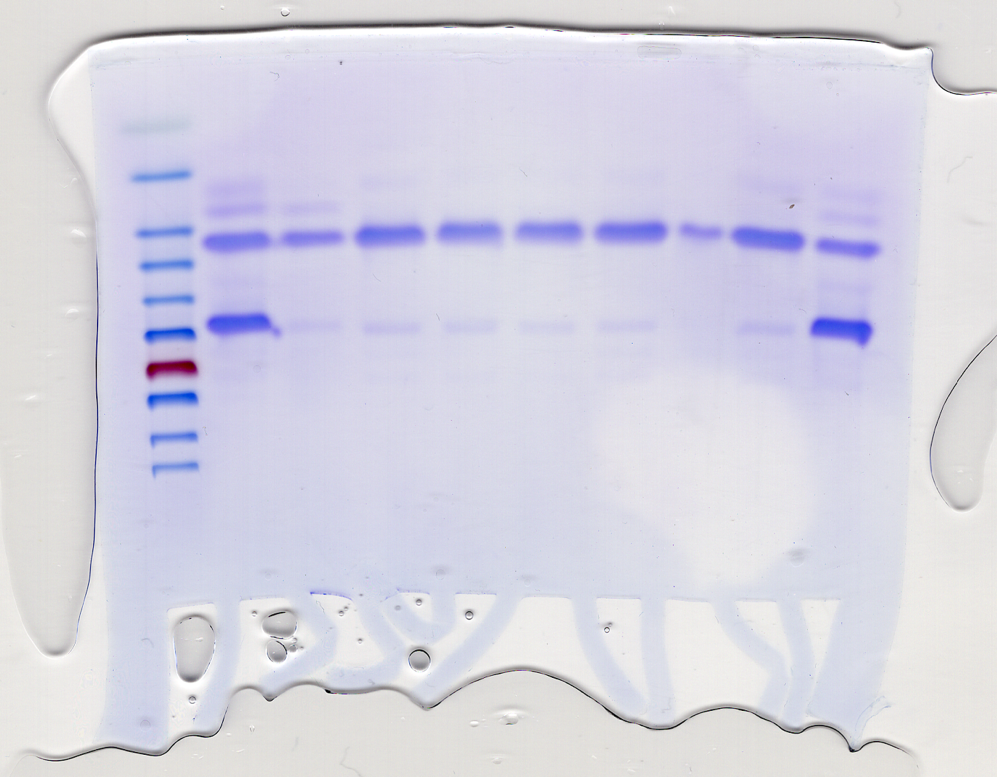
**

**
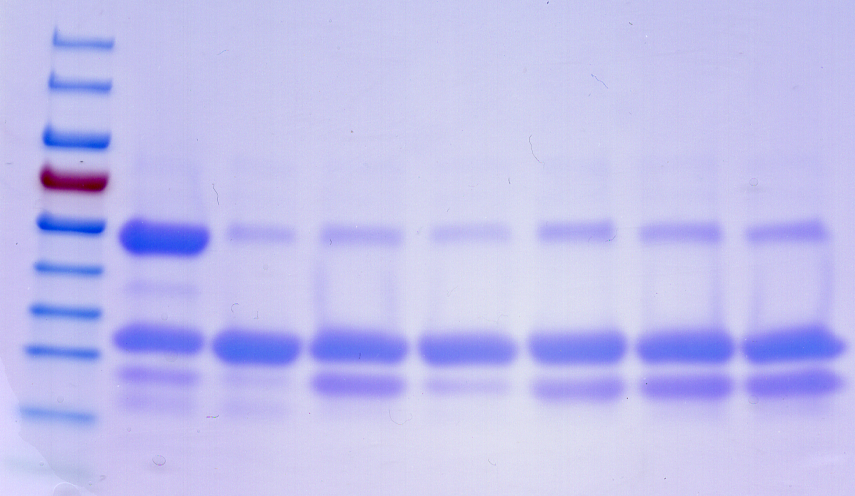
**
